# Causal association between celiac disease and inflammatory bowel disease: A two-sample bidirectional Mendelian randomization study

**DOI:** 10.3389/fimmu.2022.1057253

**Published:** 2023-01-04

**Authors:** Shuai Yuan, Ji Hun Kim, Pai Xu, Zhao Wang

**Affiliations:** ^1^ Division of Pancreatobiliary Surgery, Department of Surgery, Ajou University School of Medicine, Suwon, Republic of Korea; ^2^ Department of Orthopaedic Surgery, Chungnam National University School of Medicine, Daejeon, Republic of Korea

**Keywords:** inflammatory bowel disease, celiac disease, Crohn’s disease, ulcerative colitis, mendelian randomization

## Abstract

**Background:**

An epidemiological link between celiac disease (CeD) and inflammatory bowel disease (IBD) has been well established recently. In this study, Mendelian randomization (MR) analysis was performed employing pooled data of publicly available genome-wide association studies (GWAS) to determine the causal relationship between CeD and IBD, encompassing ulcerative colitis (UC) and Crohn’s disease (CD).

**Methods:**

Dataset of CeD was acquired from GWAS for 12,041 cases and 12,228 controls. A GWAS of more than 86,000 patients and controls was used to identify genetic variations underlying IBD. MR analyses were performed with an inverse-variance-weighted approach, an MR-Egger regression, a weighted-mode approach, a weighted-median method, and sensitivity analyses of MR pleiotropy residual sum and outlie (MR-PRESSO).

**Results:**

MR demonstrated that genetic predisposition to CeD was linked to a augmented risk of IBD (OR: 1.1408; 95% CI: 1.0614-1.2261; *P* = 0.0003). In the analysis of the two IBD subtypes, genetic predisposition to CeD was also linked to increased risks of UC (OR: 1.1646; 95% CI: 1.0614-1.2779; *P* = 0.0012) and CD (OR: 1.1865; 95% CI: 1.0948-1.2859; P = 3.07E-05). Reverse MR analysis results revealed that genetic susceptibility to IBD and CD was correlated with an augmented risk of CeD. However, there was no genetic correlation between UC and CeD. All of the above results were validated with other GWAS databases.

**Conclusion:**

There is a bidirectional causal relationship of CeD with IBD and CD. However, UC only augments the risk of developing CeD.

## Introduction

IBD including CD and UC is characterized by chronic recurring inflammation of the gastrointestinal tract. It has a significant sickness burden globally (1, 2). CD can impact any portion of the gastrointestinal tract, with the colon and terminal ileum being the most commonly impacted sites. Inflammation in CD is discontinuous (i.e., skip areas) and frequently transmural. UC primarily affects the rectum and a portion of the colon, spreading in a continuous pattern. It is limited to the mucosa. It has more superficial inflammation than CD (3, 4). Celiac disease (CeD) is a chronic intestinal ailment associated with an immune system disorder and gluten intolerance. It causes parenteral and gastrointestinal symptoms in about 1.4% of the global population (5). Despite the fact that genetics contribute to the development of CeD, new evidence from observational research has shown that CeD is strongly linked to other chronic diseases, implying common causes or risk factors (5, 6). CeD and IBD are inflammatory gastrointestinal diseases that are increasing in incidence and prevalence. Both diseases are thought to share some genetic, immune, and environmental factors that either directly cause the disease or affect other factors to cause the disease, resulting in an altered immune response in patients with a genetic tendency (7–10).

The relationship between IBD and CeD has recently piqued people’s interest. Both illnesses are believed to be related to the interplay of specific environmental elements, which either cause the illness directly or make other factors cause the sickness and induce an altered immune response in patients with a hereditary predisposition (11). According to some studies, the risk of getting IBD is 5-10 times higher in CeD cases than in the general population (7, 12), while the risk of CeD in IBD patients is only moderately higher (13). However, other research have found that the risk of getting CeD is not augmented in IBD patients (12, 14). A recent meta-analysis (15) involving 65 studies has discovered a bidirectional correlation between CeD and IBD (including CD and UC). In patients with CeD, the risk of IBD was augmented 9-fold and the risk of CD was higher than that of UC. In cases with IBD, the risk of CeD was moderately increased and the risk of CD was higher than that of UC (15). Although increasing evidence (16, 17) has shown that CeD and IBD might influence each other, whether these diseases are causally related to each other remains unclear. At the same time, the majority of earlier research was observational and cannot be used to establish causation because it could have confounding variables and reverse causality (18).

MR is a method for determining genetic variations. By combining GWAS data, MR can effectively reduce results’ deviation caused by confounding and reverse causality of genetic variation as a tool variable (19). MR is frequently used to ascertain if exposure and outcomes are causally related. If the exposure is causal, instrumental variables that affect the exposure will have proportional effects on results (20). In this study, MR analysis was performed for two-sample GWAS data to evaluate the causal correlation between CeD and IBD (encompassing CD and UC).

## Methods

### Research and data sources

**Figure 1:**
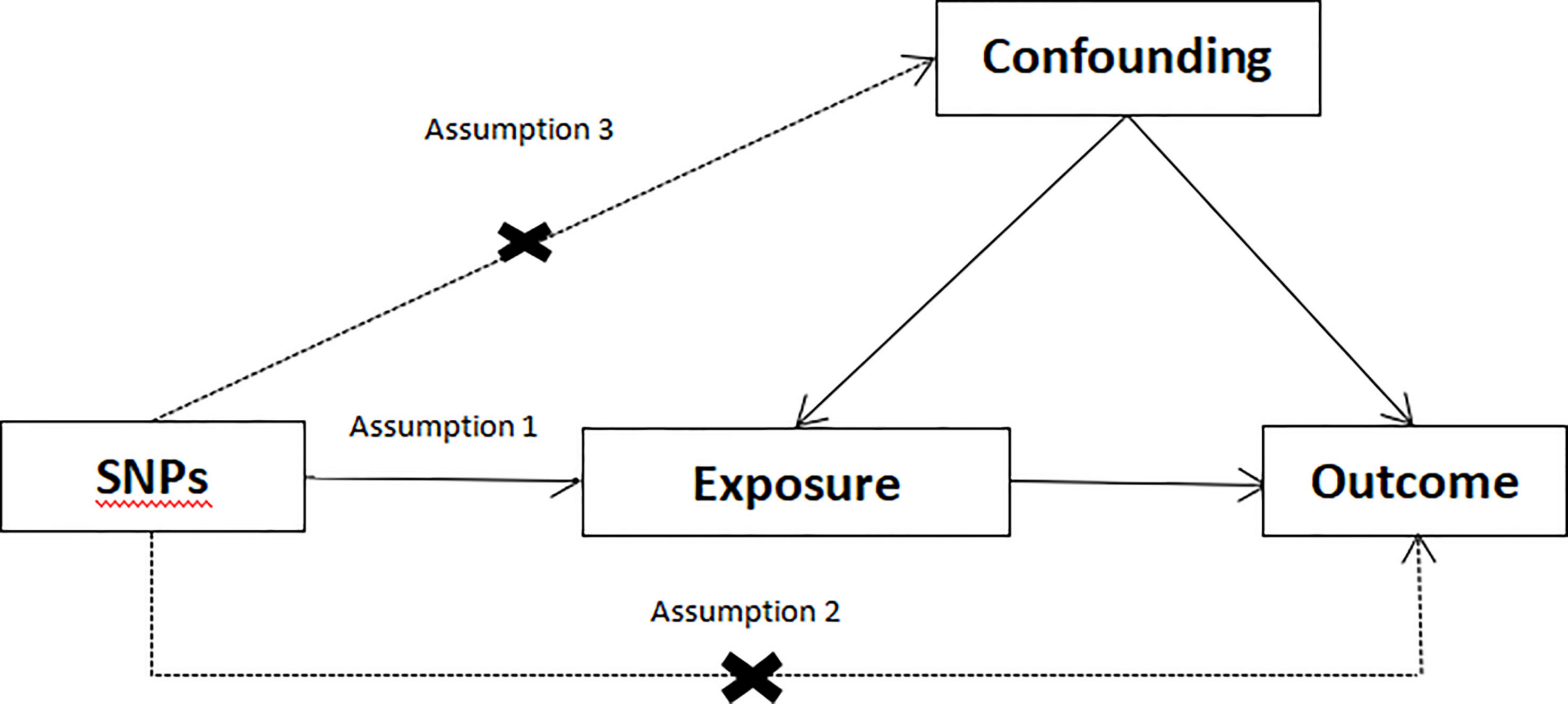
Conceptual framework diagram for Mendelian randomization analysis.

In MR research, the instrumental variable (IV) must meet three fundamental assumptions (**Figure 1**) (1): genetic variation ought to be significantly relevant to exposure; (2) genetic variation ought to be linked to exposure, not linked to any confounding factors associated with the outcome. (3) genetic variation should have nothing to do with exposure or confounding factor-dependent outcome. It is difficult to estimate causality without making any of the above assumptions (21). We used data from published research or publicly available GWAS statistics.

The biggest GWAS reported for IBD (encompassing UC and CD) to date was selected to produce more complete and reliable results (22). Another GWAS study of IBD (23) was also included for verification. SNPs linked to IBD (encompassing CD and UC) were extracted. Effect estimation of SNPs relevant to CeD was extracted from two GWAS published CeD databases (24, 25). Dataset details are listed in **Table 1**. To avoid pleiotropic deviation of cross-lineage cases (26), all research participants were of European ancestry.

**Table 1:** Description of GWAS summary samples used in this study.

Trait	Sample size	Number of SNPs	Populations	Reference
CeD	23,649	97,422	European	24
CeD	15,283	518,292	European	25
IBD	34,652	12,716,084	European	22
UC	27,432	12,255,197	European	22
CD	20,883	12,276,506	European	22
IBD	75,000	14.378	European	23
UC	26,897	10,662	European	23
CD	30,740	13,898	European	23

GWAS, Genome Wide Association Study; SNP, single nucleotide polymorphism; CeD, Celiac Disease; IBD, Inflammatory Bowel Disease; UC, Ulcerative Colitis; CD, Crohn’s Disease.

### Selection of instrumental variables

The best instrumental variables were chosen using the following high-quality procedures to guarantee the integrity and precision of study findings. Firstly, SNP (*P* < 5×10^-8^) known to significantly related to IBD with a genome-wide significance was selected as an instrumental variable. Secondly, palindromic SNPs with minor allele frequency (MAF) threshold of 0.3 were allowed. Thirdly, one of the principles of MR method was that there was no linkage disequilibrium (LD) clumping among included instrumental variables because the existence of strong LD might lead to deviation of results. In the present study, we selected independent SNPs (r2<0.001 and distance >10000kb) using the “clump”. Fourthly, it was critical in MR to ensure that the influence of SNP on exposure corresponded to the same allele as the influence on results. This principle states that palindrome SNP will not be included in instrumental variables. These carefully chosen SNPs served as instrumental variables in subsequent MR analysis. Our study investigated and excluded pleiotropic SNPs linked to confounding variables connected to exposure-outcome using the PhenoScanner database to supplement the evidence (27, 28). The selected SNP ought to be closely relevant to exposure according to the MR analysis hypothesis. F-statistic is widely used to gauge how strongly instrumental variables are related to exposure. According to previous research, instrumental variables and exposure have a weak correlation if the F-statistic is more than 10 (29).

### Mendelian randomization analysis

The main analysis for MR is inverse variance weighting (IVW) (30), which is essentially a meta-analysis method. To generate an overall estimate of the influence of exposure on outcome, it is translated into a weighted regression of the impact of instrumental factors on outcomes of exposure effects. IVW can avoid confounding variables without a horizontal pleiotropy and produce unbiased estimation. Furthermore, we employed the following three additional methods that permitted horizontal pleiotropy with a lower statistical capability than IVW: (1) the MR-Egger regression (31); (2) the weighted median method (32); and (3) the weighted mode method (33).

### Sensitivity analysis

According to MR, the genetic instrument can only influence the result by exposing people to it. Gene variations might have pleiotropic effects. Estimates may be inaccurate if the SNPs used as instruments have a horizontal pleiotropic impact, which causes the outcome to be influenced by a pathway other than the exposure. Using more SNPs might prevent this bias if the pleiotropic effects are balanced. Bias is also less likely by estimates consistent across numerous methodologies and various pleiotropy assumptions. Outliers in IVW linear regression might be detect and correct using the MR-PRESSO (34). To pass the MR-PRESSO outlier test, a minimum of 50% of variations should be genuine instrumental variables, should have balanced pleiotropy, and should depend on the instrumental strength independent of direct effects (InSIDE) condition. Furthermore, the heterogeneity of the chosen SNPs was evaluated employing the Cochran Q test (35). This study employed Cochran Q analysis to assess heterogeneity and considered the fixed-effects IVW approach as the main approach if *p*-values were higher than 0.05 without evidence of heterogeneity. The random-effects IVW approach was utilized if there was substantial heterogeneity (*p* < 0.05). R software (version 4.0.2, MR package) was employed to perform all statistical analyses.

## Results

The genetic correlation between CD and UC (two subtypes of IBD) was 19.5%. These two subtypes rarely coexist due to their limited genetic link.

### Causal relationship between CeD and IBD

According to the above selection standard, the linkage disequilibrium test was conducted to select SNPs associated with CeD and IBD (including UC and CD) at first. SNPs with F-statistics below 10 were then removed. Confounding factors were removed based on the PhenoScanner Database. Finally, abnormal values were removed through MR-PRESSO global outlier test. A total of 23, 29, and 26 SNPs related to CeD in association with IBD, UC, and CD, respectively, were obtained (**Supplemental Table S1**).

**Figure 2:**
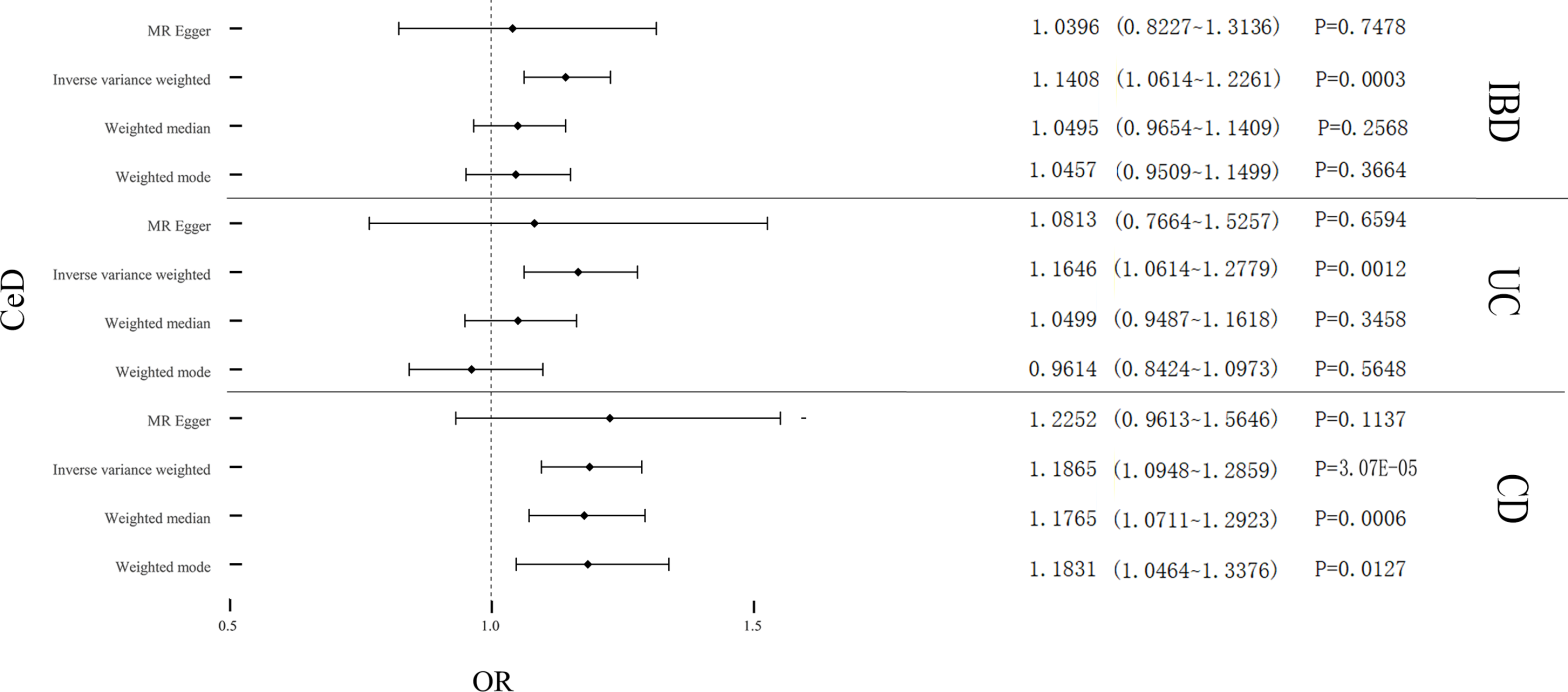
Causal estimates given as odds ratios (ORs) and 95% confidence intervals for the effect of CeD on IBD, UC, and CD. CeD, Celiac Disease; IBD, Inflammatory Bowel Disease; UC, Ulcerative Colitis; CD, Crohn’s Disease.

Cochran’s Q test revealed significant heterogeneity (P_IBD_ = 0.002; P_UC_ = 6.48E-07; P_CD_ = 0.008) (**Table 2**). Analyses were conducted using the IVW with the multiplicative random-effects model. After pleiotropic SNPs were removed (**Supplemental Table S5**), a positive correlation between CeD and IBD (predicted genetically) was found (OR: 1.1408; 95% CI: 1.0614-1.2261; *P* = 0.0003). Analysis of the two IBD subtypes revealed that CeD was linked to both UC (OR: 1.1646; 95% CI: 1.0614-1.2779; *P* = 0.0012) and CD (OR: 1.1865; 95% CI: 1.0948-1.2859; P = 3.07E-05) (**Figure 2**). No remarkable horizontal pleiotropy was found using sensitivity analysis (regression intercept of MR-Egger was nearly zero, P_IBD_ = 0.4221; P_UC_ = 0.6641; P_CD_ = 0.7851) (**Table 2**). Funnel plot, leave-one-out analysis, scatter plot, and forest plot of MR are presented in **Supplementary Figures 1-3**.

**Table 2:** Heterogeneity and pleiotropy analysis of CeD with IBD, UC, and CD using different analytical methods.

Exposure traits	Outcome traits	MR methods	Cochran Q statistic	Heterogeneity *p*-value	Pleiotropy *p*-value	MR-PRESSO global outlier test	
						RSSOBs	*p*-value
CeD	IBD	IVW	45.846	0.002	0.422	50.4317	0.002
	UC	IVW	80.0846	6.48E-07	0.664	87.7359	<0.001
	CD	IVW	44.964	0.008	0.785	48.2256	0.011

MR, Mendelian Randomization; CeD, Celiac Disease; CD, Crohn’s Disease; MR-PRESSO, MR pleiotropy residual sum and outlier; IBD, Inflammatory Bowel Disease; UC, Ulcerative Colitis; IVW, Inverse variance weighted.

We conducted a verification test using another GWAS database and obtained 8, 8, and 9 SNPs related to CeD with IBD, UC, and CD in the same way (**Supplemental Table S2**). Cochran’s Q test revealed significant heterogeneity (P_IBD_ = 0.0001; P_UC_ = 0.0056; P_CD_ = 0.0141) (**Supplemental Table S9**). Analyses were conducted using the IVW with the multiplicative random-effects model. After pleiotropic SNPs were removed (**Supplemental Table S6**), a positive correlation between CeD and IBD (predicted genetically) was found (OR: 1.2738; 95% CI: 1.1176-1.4519; *P* = 0.0002). Analysis two subtypes of IBD, CeD was associated with UC (OR: 1.2992; 95% CI: 1.1404-1.4801; *P* = 8.31E-05) and CD (OR: 1.2804; 95% CI: 1.1343-1.4453; *P* = 6.34E-05) (**Supplemental Figure 13**). No remarkable horizontal pleiotropy was found using sensitivity analysis (P_IBD_ = 0.6949; P_UC_ = 0.3858; P_CD_ = 0.3637) (**Supplemental Table S9**). Funnel plot, leave-one-out analysis, scatter plot, and forest plot of MR analysis are presented in **Supplementary Figures 4-6**. The conclusion was still suitable for the verification group. Thus, a positive link between genetic susceptibility of CeD and risk of IBD (encompassing UC and CD) was discovered in MR.

### Causal relationship between IBD and CeD

According to the same selection standard, the linkage disequilibrium test was conducted to select SNPs linked to IBD (encompassing UC and CD) and CeD at first. SNPs with F-statistics below 10 were then removed. Confounding factors were removed based on the PhenoScanner Database. Finally, abnormal values were removed through MR-PRESSO global outlier test. A total of 21, 13 and 15 SNPs related to IBD, UC, and CD, respectively, in association with CeD were obtained (**Supplemental Table S3**).

**Figure 3:**
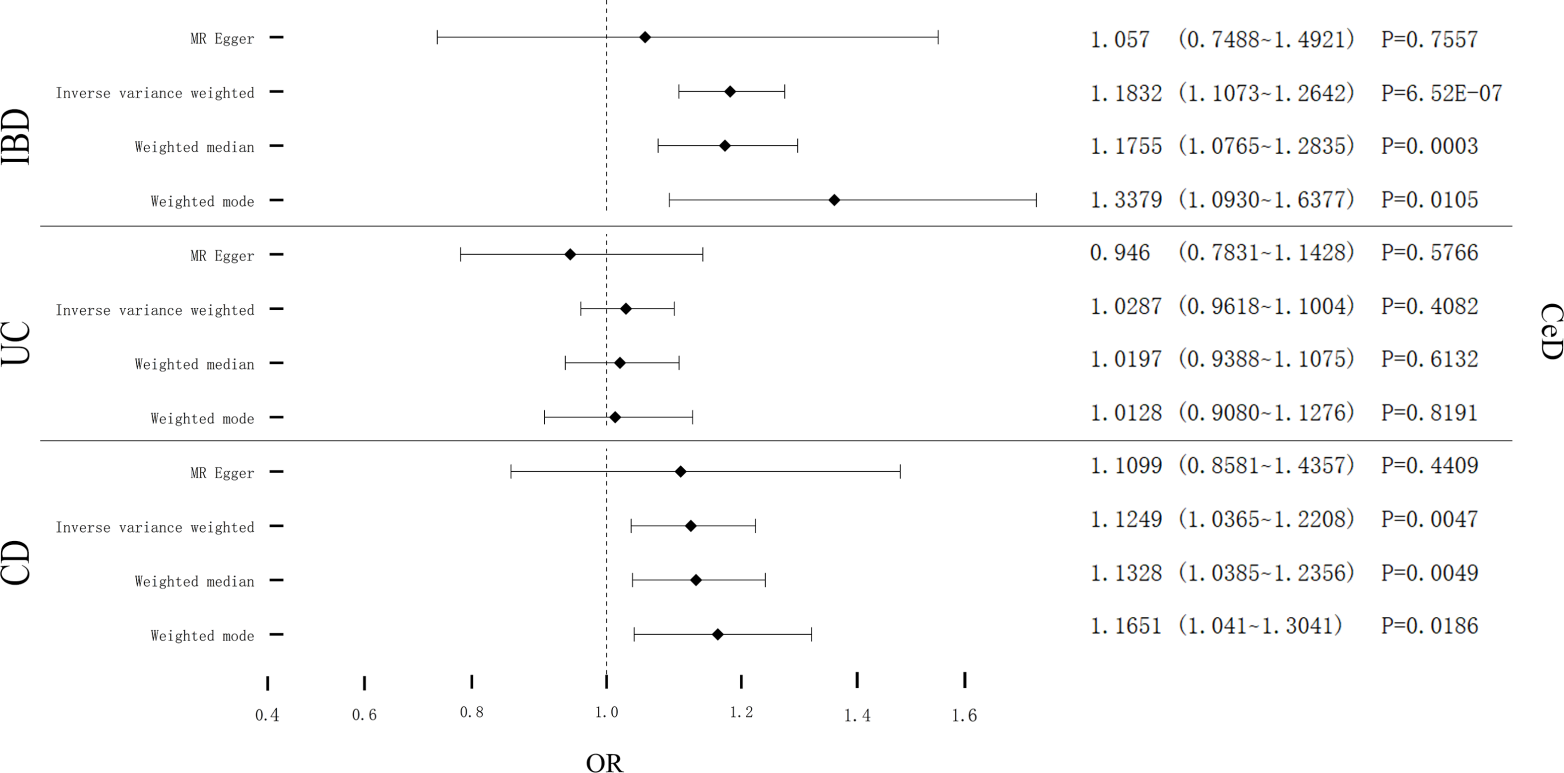
Causal estimates given as odds ratios (ORs) and 95% confidence intervals for the effect of IBD, UC, and CD on CeD. IBD, Inflammatory Bowel Disease; UC, Ulcerative Colitis; CD, Crohn’s Disease; CeD, Celiac Disease.

Cochran’s Q test showed remarkable heterogeneity (P_IBD_ = 0.1201; P_UC_ = 0.1997; P_CD_ = 0.0035) (**Table 3**). Therefore, analyses were conducted using the IVW with the multiplicative fixed-effects model for IBD and UC. Analyses were conducted using the IVW with the multiplicative random-effects model for CD. After pleiotropic SNPs were removed (**Supplemental Table S7**), a positive correlation between CeD and IBD (predicted genetically) was found (OR: 1.1832; 95% CI: 1.1073-1.2642; *P* = 6.52E-07). However, different results were obtained in the analysis for the two subtypes of IBD, showing no genetic correlation between UC and CeD (OR: 1.0287; 95% CI: 0.9618-1.1004; *P* = 0.4082). CD had a positive correlation with CeD (OR: 1.1249; 95% CI: 1.0365-1.2208; *P* = 0.0047) (**Figure 3**). No remarkable horizontal pleiotropy was discovered using sensitivity analysis (P_IBD_ = 0.5212; P_UC_ = 0.3715; P_CD_ = 0.9157) (**Table 3**). Funnel plot, leave-one-out analysis, scatter plot, and forest plot of MR are presented in **Supplementary Figures 7-9**.

**Table 3:** Heterogeneity and pleiotropy analysis of IBD, UC, and CD with CeD using different analytical methods.

Exposure traits	MR methods	CeD				
		Cochran Q statistic	Heterogeneity *p*-value	Pleiotropy *p*-value	MR-PRESSO global outlier test	
					RSSOBs	*p*-value
IBD	IVW	27.5626	0.1201	0.5212	30.9557	0.125
UC	IVW	15.8174	0.1997	0.3715	18.3589	0.212
CD	IVW	32.4019	0.0035	0.9157	36.8474	0.01

MR, Mendelian Randomization; CeD, Celiac Disease; UC, Ulcerative Colitis; MR-PRESSO, MR pleiotropy residual sum and outlier CD, Crohn’s Disease; IBD, Inflammatory Bowel Disease; IVW, Inverse variance weighted.

We also conducted a verification test using another GWAS database and obtained 55, 43, and 47 SNPs related to IBD, UC, and CD, respectively, in association with CeD (**Supplemental Table S4**). Cochran’s Q test showed remarkable heterogeneity (P_IBD_ = 6.22E-07; P_UC_ = 0.0176; P_CD_ = 0.0008) (**Supplemental Table S10**). Analyses were conducted using the IVW with the multiplicative random-effects model. After pleiotropic SNPs were removed (**Supplemental Table S8**), a positive correlation between CeD and IBD (predicted genetically) was found (OR: 1.2117; 95% CI: 1.1336-1.2953; *P* = 1.61E-08). Analysis results for the two subtypes of IBD were the same as those for the total experimental group. There was no genetic correlation between UC and CeD (OR: 1.0276; 95% CI: 0.9639-1.0893; *P* = 0.3598). There was a positive correlation between CD and CeD (OR: 1.1465; 95% CI: 1.0885-1.2076; *P* = 2.46E-07) (**Supplemental Figure 14**). No remarkable horizontal pleiotropy was found using sensitivity analysis (P_IBD_ = 0.1061; P_UC_ = 0.1823; P_CD_ = 0.1302) (**Supplemental Table S10**). Funnel plot, leave-one-out analysis, scatter plot, and forest plot of MR are presented in **Supplementary Figures 10-12**. Conclusion obtained for the experimental group was still suitable for the verification group. Thus, there is a positive link between genetic susceptibility of IBD and risk of CeD. However, UC and CD play different roles in CeD. There is a positive link between genetic susceptibility of CD and the risk of CeD, and UC will not augment the risk of CeD.

## Discussion

Within this MR analysis employing the GWAS study database, we assessed the causal link between CeD and IBD. What we discovered was that genetic susceptibility to CeD was linked to augmented IBD, CD, and UC risks. Similarly, genetic susceptibility to IBD was linked to an augmented risk of CeD. The association with CeD showed a difference between CD and UC. CD raised the risk of developing CeD. However, UC did not raise the risk of developing CeD. Therefore, there is a bidirectional causal effect for the association of CeD with IBD and CD, whereas UC only increases the risk of developing CeD.

Evidence from observational studies suggests that IBD and CeD often occur together (7, 15, 36, 37). Several meta-analyses and reviews have found that cases with IBD have an intermediate risk of linked CeD (3.96; 95% CI: 2.23-7.02) (13, 15). Contrarily, cases with CeD have a reported prevalence of IBD of 1-3.2% (7, 14, 38), which is 3–10 times higher than the prevalence of IBD in the general population, indicating that cases with CeD are significantly more likely to develop IBD (38, 39). The significantly higher risk of developing IBD in CeD cases has also been demonstrated by Matteo et al. (36) who have also compared subtypes CD and UC, further indicating that CeD cases have a considerably higher risk of getting IBD, CD, and UC. A recent large meta-analysis study (15) with 43,026 CeD cases, 165,637 IBD cases (38,606 with CD and 55,515 with UC), and 13,470,350 controls has discovered that relative risks of developing IBD, CD, and UC in CeD patients are 2.90 (95% CI: 1.88-4.48), 3.15 (95% CI: 1.77-5.62), and 2.81 (95% CI: 1.82-4.36), respectively. In contrast to controls, CeD was also observed in cases with IBD, CD, and UC with relative risks of 5.32 (95% CI: 3.79-7.46); 7.73 (95% CI: 5.09-11.73), and 4.08 (95% CI: 2.40-6.95), respectively. These investigations back up the current finding that there is a link between IBD and CeD. However, other studies have shown the potential of worse outcomes when IBD and CeD co-exist. Oxford et al. (40) have found that cases with both UC and CeD are more inclined to have complete colitis than controls in a study of 51 cases with concurrent IBD and CeD (OR: 3.30; 95% CI: 1.05-21.50). There was a higher frequency of IBD in 455 cases with CeD in a retrospective research by Yang et al. (14) In addition, three out of five cases with combined UC and CeD needed a colectomy for refractory UC. These investigations have provided a theoretical basis for further research into the long-term prognosis of patients with co-existing CeD and IBD.

At present, evidence suggests that CeD and IBD might share genetic loci. For example, 70% (113/163) and 12% (20/163) of IBD loci are shared with other complicated disorders and CeD, respectively. While using loci in the CeD risk region as a reference, 50% of CeD loci were shared with IBD (37). Patients with CeD had a substantially higher risk of getting IBD than those without CeD. By contrast, there was only a small augment in the prevalence of CeD with IBD compared to population controls (13, 41). This association might be due to shared genetic risk factors for the disorder. In meta-analysis, Festen et al. (41) have identified four common risk loci (IL18RAP, PTPN2, TAGAP, and, PUS10) between CeD and CD, suggesting that there might be a common genetic pathway for the development of these two disorders. Furthermore, the association between CeD and CD can be explained by intraepithelial T cells, a key point responsible for immunopathogenesis of both disorders. Some autoantibodies have been noted in patients with CeD and IBD (12). For instance, 39%-70% of CD patients were positive for anti-*Saccharomyces cerevisiae* antibodies (ASCAs) (42). In CeD, the ASCA positivity rate was 67% (43), suggesting a correlation between CeD and CD, which might explain the bidirectional causality of CeD and CD. In one study by Snook et al. (44), the positivity rate for antinuclear antibodies (ANAs) was 25%-51% in UC and 8%-17% in CeD. The HLA region can be observed as an association signal for most immune-mediated diseases. The impact of the HLA region is different for IBD and CeD, with the HLA locus being the major genetic susceptibility factor for CeD, accounting for 40% of the genetic risk. However, the HLA alleles associated with IBD are moderately associated and influence the genetic risk of IBD (23, 45). In addition to the HLA region, 163 loci were linked to IBD risk, and 40 susceptibility loci were linked to CeD risk (46). Although these data provide a correlation between the genetic basis of IBD and CeD, further validation and studies are needed for the genetic relationship between the two diseases. These data indicate that there is an association between CeD and UC. However, they are insufficient to draw conclusions about the causality. To activate the immune system and lead to pathological processes, IBD and CeD both require an environmental stimulus. Recent findings have demonstrated that IBD and CeD have extensive overlaps in their immune-mediated basis and genetic basis (37). A bidirectional association between IBD and CeD is also plausible. However, further research is needed on their causal relationship.

The clinical presentation of CeD and IBD are similar (47), and patients with CeD are often investigated for the presence of IBD, as the two disorders may coexist. However, not all patients diagnosed with CeD by serology and histology receive a combined diagnosis of IBD at the time of diagnosis, and vice versa (48). Therefore, this study provides a theoretical basis for the coexistence of both diseases, making clinicians pay more attention to diagnosing and preventing patients with CeD and IBD (encompassing UC and CD) in clinical practice.

Our study is the first two-sample MR analysis of IBD and CeD. MR is less vulnerable to non-differential measurement error, reverse causation, and confounding than observational research. The iterative method is conservative. It confirms the uniformity of the point calculated before and after removing outliers, strengthening the evidence. Additionally, our study also performed additional sensitivity analyses to ensure the consistency of causal estimates and to test the robustness of this study’s results.

Our study has several limitations. Firstly, participants from the exposure and result studies included in the MR ought to not overlap. However, we could not determine the extent of overlap in this study. Secondly, there is a limit in generalizing current results other races because results of this study are based on people of European ancestry. Therefore, caution is needed when using our findings in racially and ethnically diverse populations. Thirdly, there are strengths and weaknesses to each of the methods we used in our analysis. However, using different methods based on various presumptions could raise the likelihood of obtaining inconsistent or opposing results and obscure conclusions. Fourthly, due to the limitations of the GWAS summary statistics, the MR based on different ages, gender, and height was not feasible. Fifthly, the analysis divided pleiotropy into vertical and horizontal pleiotropy; the presence of horizontal pleiotropy violates the MR’s presumptions and introduces bias, which significantly impacts the study’s findings. However, there was no horizontal pleiotropy in the Egger intercept of the MR, demonstrating that the investigation of pleiotropy can successfully eliminate bias and increase the stability of results. Sixthly, even if confounding has been removed in this study, the influence of third-party conditions cannot be excluded, so the results may be nonlinear and need further confirmation.

## Conclusions

We confirmed a bidirectional causal effect link of CeD with IBD and CD. We found that UC only increased the risk of developing CeD. This gives more weight to the diagnosis and prevention of CeD patients and IBD (encompassing UC and CD) patients in clinical practice. It also provides a new direction for research into both CeD and IBD (encompassing UC and CD).

## Data availability statement

Publicly available datasets can be found here: the Integrative Epidemiology Unit (IEU) GWAS database (https://gwas.mrcieu.ac.uk).

## Ethics statement

Because this study was based on data at the summary level, ethics approval and participant consent were acquired during the initial research.

## Author contributions

SY and JK conceived the idea for the study. SY, PX, and ZW obtained genetic data. SY and PX performed data analyses. SY and JK interpreted results of data analyses. SY and PX drafted the manuscript. JK reviewed the final manuscript. All authors read and approved the final manuscript.

## References

[1] Hodson R. Inflammatory bowel disease. Nature (2016) 540(7634):S97. doi: 10.1038/540S97a 28002398

[2] Rosen MJ Dhawan A Saeed SA. Inflammatory bowel disease in children and adolescents. JAMA Pediatr (2015) 169(11):1053–60. doi: 10.1001/jamapediatrics.2015.1982 PMC4702263 26414706

[3] Khor B Gardet A Xavier RJ. Genetics and pathogenesis of inflammatory bowel disease. Nature (2011) 474(7351):307–17. doi: 10.1038/nature10209 PMC3204665 21677747

[4] Abraham C Cho JH. Inflammatory bowel disease. N Engl J Med (2009) 361(21):2066–78. doi: 10.1056/NEJMra0804647 PMC3491806 19923578

[5] Kivela L Caminero A Leffler DA Pinto-Sanchez MI Tye-Din JA Lindfors K. Current and emerging therapies for coeliac disease. Nat Rev Gastroenterol Hepatol (2021) 18(3):181–95. doi: 10.1038/s41575-020-00378-1 33219355

[6] Tang Z Shen M Chen X Liu Y. Association between transferrin saturation and celiac disease: A two-sample mendelian randomization study. Pediatr Allergy Immunol (2021) 32(7):1575–7. doi: 10.1111/pai.13565 34028910

[7] Kocsis D Toth Z Csontos AA Miheller P Pak P Herszenyi L. Prevalence of inflammatory bowel disease among coeliac disease patients in a Hungarian coeliac centre. BMC Gastroenterol (2015) 15:141. doi: 10.1186/s12876-015-0370-7 26481725 PMC4612406

[8] Bosca-Watts MM Minguez M Planelles D Navarro S Rodriguez A Santiago J. Hla-dq: Celiac disease vs inflammatory bowel disease. World J Gastroenterol (2018) 24(1):96–103. doi: 10.3748/wjg.v24.i1.96 29358886 PMC5757130

[9] West J Fleming KM Tata LJ Card TR Crooks CJ. Incidence and prevalence of celiac disease and dermatitis herpetiformis in the uk over two decades: Population-based study. Am J Gastroenterol (2014) 109(5):757–68. doi: 10.1038/ajg.2014.55 PMC4012300 24667576

[10] Fernandez A Gonzalez L de-la-Fuente J. Coeliac disease: Clinical features in adult populations. Rev Esp Enferm Dig (2010) 102(8):466–71. doi: 10.4321/s1130-01082010000800002 20670066

[11] Festen EA Szperl AM Weersma RK Wijmenga C Wapenaar MC. Inflammatory bowel disease and celiac disease: Overlaps in the pathology and genetics, and their potential drug targets. Endocr Metab Immune Disord Drug Targets (2009) 9(2):199–218. doi: 10.2174/187153009788452426 19519468

[12] Bengi G Civak M Akarsu M Soyturk M Ellidokuz E Topalak O. Prevalance of celiac disease in patients with inflammatory bowel disease in Turkish population. Gastroenterol Res Pract (2019) 2019:6272098. doi: 10.1155/2019/6272098 31885543 PMC6927052

[13] Shah A Walker M Burger D Martin N von Wulffen M Koloski N. Link between celiac disease and inflammatory bowel disease. J Clin Gastroenterol (2019) 53(7):514–22. doi: 10.1097/MCG.0000000000001033 29762265

[14] Yang A Chen Y Scherl E Neugut AI Bhagat G Green PH. Inflammatory bowel disease in patients with celiac disease. Inflammation Bowel Dis (2005) 11(6):528–32. doi: 10.1097/01.mib.0000161308.65951.db 15905699

[15] Pinto-Sanchez MI Seiler CL Santesso N Alaedini A Semrad C Lee AR. Association between inflammatory bowel diseases and celiac disease: A systematic review and meta-analysis. Gastroenterology (2020) 159(3):884–903.e31. doi: 10.1053/j.gastro.2020.05.016 32416141

[16] Grode L Bech BH Jensen TM Humaidan P Agerholm IE Plana-Ripoll O. Prevalence, incidence, and autoimmune comorbidities of celiac disease: A nation-wide, population-based study in Denmark from 1977 to 2016. Eur J Gastroenterol Hepatol (2018) 30(1):83–91. doi: 10.1097/MEG.0000000000000992 29076940

[17] Conti L Lahner E Galli G Esposito G Carabotti M Annibale B. Risk factors associated with the occurrence of autoimmune diseases in adult coeliac patients. Gastroenterol Res Pract (2018) 2018:3049286. doi: 10.1155/2018/3049286 30275824 PMC6157138

[18] Greenland S Robins JM. Confounding and misclassification. Am J Epidemiol (1985) 122(3):495–506. doi: 10.1093/oxfordjournals.aje.a114131 4025298

[19] Emdin CA Khera AV Kathiresan S. Mendelian randomization. JAMA (2017) 318(19):1925–6. doi: 10.1001/jama.2017.17219 29164242

[20] Davies NM Holmes MV Davey Smith G. Reading mendelian randomisation studies: A guide, glossary, and checklist for clinicians. BMJ (2018) 362:k601. doi: 10.1136/bmj.k601 30002074 PMC6041728

[21] Lawlor DA Harbord RM Sterne JA Timpson N Davey Smith G. Mendelian randomization: Using genes as instruments for making causal inferences in epidemiology. Stat Med (2008) 27(8):1133–63. doi: 10.1002/sim.3034 17886233

[22] Liu JZ van Sommeren S Huang H Ng SC Alberts R Takahashi A. Association analyses identify 38 susceptibility loci for inflammatory bowel disease and highlight shared genetic risk across populations. Nat Genet (2015) 47(9):979–86. doi: 10.1038/ng.3359 PMC4881818 26192919

[23] Jostins L Ripke S Weersma RK Duerr RH McGovern DP Hui KY. Host-microbe interactions have shaped the genetic architecture of inflammatory bowel disease. Nature (2012) 491(7422):119–24. doi: 10.1038/nature11582 PMC3491803 23128233

[24] Trynka G Hunt KA Bockett NA Romanos J Mistry V Szperl A. Dense genotyping identifies and localizes multiple common and rare variant association signals in celiac disease. Nat Genet (2011) 43(12):1193–201. doi: 10.1038/ng.998 PMC3242065 22057235

[25] Dubois PC Trynka G Franke L Hunt KA Romanos J Curtotti A. Multiple common variants for celiac disease influencing immune gene expression. Nat Genet (2010) 42(4):295–302. doi: 10.1038/ng.543 20190752 PMC2847618

[26] Burgess S Davey Smith G Davies NM Dudbridge F Gill D Glymour MM. Guidelines for performing mendelian randomization investigations. Wellcome Open Res (2019) 4:186. doi: 10.12688/wellcomeopenres.15555.2 32760811 PMC7384151

[27] Kamat MA Blackshaw JA Young R Surendran P Burgess S Danesh J. Phenoscanner V2: An expanded tool for searching human genotype-phenotype associations. Bioinformatics (2019) 35(22):4851–3. doi: 10.1093/bioinformatics/btz469 PMC6853652 31233103

[28] Staley JR Blackshaw J Kamat MA Ellis S Surendran P Sun BB. Phenoscanner: A database of human genotype-phenotype associations. Bioinformatics (2016) 32(20):3207–9. doi: 10.1093/bioinformatics/btw373 PMC5048068 27318201

[29] Pierce BL Ahsan H Vanderweele TJ. Power and instrument strength requirements for mendelian randomization studies using multiple genetic variants. Int J Epidemiol (2011) 40(3):740–52. doi: 10.1093/ije/dyq151 PMC3147064 20813862

[30] Lee CH Cook S Lee JS Han B. Comparison of two meta-analysis methods: Inverse-Variance-Weighted average and weighted sum of z-scores. Genomics Inform (2016) 14(4):173–80. doi: 10.5808/GI.2016.14.4.173 PMC5287121 28154508

[31] Bowden J Davey Smith G Burgess S. Mendelian randomization with invalid instruments: Effect estimation and bias detection through egger regression. Int J Epidemiol (2015) 44(2):512–25. doi: 10.1093/ije/dyv080 PMC4469799 26050253

[32] Bowden J Davey Smith G Haycock PC Burgess S. Consistent estimation in mendelian randomization with some invalid instruments using a weighted median estimator. Genet Epidemiol (2016) 40(4):304–14. doi: 10.1002/gepi.21965 PMC4849733 27061298

[33] Hemani G Zheng J Elsworth B Wade KH Haberland V Baird D. The Mr-base platform supports systematic causal inference across the human phenome. Elife (2018) 7:e34408. doi: 10.7554/eLife.34408 29846171 PMC5976434

[34] Verbanck M Chen CY Neale B Do R. Detection of widespread horizontal pleiotropy in causal relationships inferred from mendelian randomization between complex traits and diseases. Nat Genet (2018) 50(5):693–8. doi: 10.1038/s41588-018-0099-7 PMC6083837 29686387

[35] Bowden J Del Greco MF Minelli C Davey Smith G Sheehan N Thompson J. A framework for the investigation of pleiotropy in two-sample summary data mendelian randomization. Stat Med (2017) 36(11):1783–802. doi: 10.1002/sim.7221 PMC5434863 28114746

[36] Bramuzzo M Lionetti P Miele E Romano C Arrigo S Cardile S. Phenotype and natural history of children with coexistent inflammatory bowel disease and celiac disease. Inflammation Bowel Dis (2021) 27(12):1881–8. doi: 10.1093/ibd/izaa360 33452803

[37] Pascual V Dieli-Crimi R Lopez-Palacios N Bodas A Medrano LM Nunez C. Inflammatory bowel disease and celiac disease: Overlaps and differences. World J Gastroenterol (2014) 20(17):4846–56. doi: 10.3748/wjg.v20.i17.4846 PMC4009516 24803796

[38] Leeds JS Horoldt BS Sidhu R Hopper AD Robinson K Toulson B. Is there an association between coeliac disease and inflammatory bowel diseases? a study of relative prevalence in comparison with population controls. Scand J Gastroenterol (2007) 42(10):1214–20. doi: 10.1080/00365520701365112 17918008

[39] Gatti S Lionetti E Balanzoni L Verma AK Galeazzi T Gesuita R. Increased prevalence of celiac disease in school-age children in Italy. Clin Gastroenterol Hepatol (2020) 18(3):596–603. doi: 10.1016/j.cgh.2019.06.013 31220637

[40] Oxford EC Nguyen DD Sauk J Korzenik JR Yajnik V Friedman S. Impact of coexistent celiac disease on phenotype and natural history of inflammatory bowel diseases. Am J Gastroenterol (2013) 108(7):1123–9. doi: 10.1038/ajg.2013.20 PMC3845216 23419379

[41] Festen EA Goyette P Green T Boucher G Beauchamp C Trynka G. A meta-analysis of genome-wide association scans identifies Il18rap, Ptpn2, tagap, and Pus10 as shared risk loci for crohn's disease and celiac disease. PloS Genet (2011) 7(1):e1001283. doi: 10.1371/journal.pgen.1001283 21298027 PMC3029251

[42] Peyrin-Biroulet L Standaert-Vitse A Branche J Chamaillard M. Ibd serological panels: Facts and perspectives. Inflammation Bowel Dis (2007) 13(12):1561–6. doi: 10.1002/ibd.20226 17636565

[43] Candelli M Nista EC Carloni E Pignataro G Rigante D Gasbarrini A. Anti-saccharomyces cerevisiae antibodies and coeliac disease. Scand J Gastroenterol (2003) 38(11):1191–2. doi: 10.1080/00365520310005523 14686725

[44] Snook JA de Silva HJ Jewell DP. The association of autoimmune disorders with inflammatory bowel disease. Q J Med (1989) 72(269):835–40. 2616728

[45] Satsangi J Welsh KI Bunce M Julier C Farrant JM Bell JI. Contribution of genes of the major histocompatibility complex to susceptibility and disease phenotype in inflammatory bowel disease. Lancet (1996) 347(9010):1212–7. doi: 10.1016/s0140-6736(96)90734-5 8622450

[46] Hunt KA Mistry V Bockett NA Ahmad T Ban M Barker JN. Negligible impact of rare autoimmune-locus coding-region variants on missing heritability. Nature (2013) 498(7453):232–5. doi: 10.1038/nature12170 PMC3736321 23698362

[47] Green PH. The many faces of celiac disease: Clinical presentation of celiac disease in the adult population. Gastroenterology (2005) 128(4 Suppl 1):S74–8. doi: 10.1053/j.gastro.2005.02.016 15825130

[48] Ludvigsson JF Bai JC Biagi F Card TR Ciacci C Ciclitira PJ. Diagnosis and management of adult coeliac disease: Guidelines from the British society of gastroenterology. Gut (2014) 63(8):1210–28. doi: 10.1136/gutjnl-2013-306578 PMC4112432 24917550

