# Supplementary material for: Causal association between celiac disease and inflammatory bowel disease: A two-sample bidirectional Mendelian randomization study
Source: Front Immunol. 2023 Jan 4;13:1057253. doi: 10.3389/fimmu.2022.1057253 (PMC9845610; doi:10.3389/fimmu.2022.1057253)
Supplement: Supplementary file 1 [file DataSheet_1.doc]

**Table S1. Characteristics of the SNPs related to Celiac Disease and Inflammatory Bowel Disease、Ulcerative colitis and Crohn's disease**

| SNP | Effects on Celiac disease | | | | | Effects on Inflammatory Bowel Disease | | | | | Chr | Position | F  statistic |
| --- | --- | --- | --- | --- | --- | --- | --- | --- | --- | --- | --- | --- | --- |
|  | EA | OA | Beta | SE | p-val | EA | OA | Beta | SE | p-val |  |  |  |
| rs1018326 | C | T | 0.151862 | 0.0185859 | 3.06E-16 | C | T | -0.0057037 | 0.017 | 0.736801 | 2 | 182007800 | 66.7 |
| rs1050976 | T | C | -0.111043 | 0.0184718 | 1.84E-09 | T | C | 0.00169856 | 0.017 | 0.9208 | 6 | 408079 | 36.1 |
| rs10790269 | T | C | 0.157239 | 0.0239751 | 5.44E-11 | T | C | 0.0238044 | 0.0215 | 0.2683 | 11 | 118729456 | 43.0 |
| rs10947460 | A | G | -0.12545 | 0.0227727 | 3.61E-08 | A | G | -0.0182964 | 0.0201 | 0.3636 | 6 | 33828234 | 30.3 |
| rs11801183 | T | C | -0.138343 | 0.0245229 | 1.69E-08 | T | C | -0.0130042 | 0.0219 | 0.5544 | 1 | 172862984 | 31.8 |
| rs11851414 | C | T | 0.120446 | 0.0220512 | 4.71E-08 | C | T | 0.0623968 | 0.0201 | 0.00194299 | 14 | 69259502 | 29.8 |
| rs12068671 | C | T | -0.156654 | 0.0244153 | 1.4E-10 | C | T | 0.00190181 | 0.0215 | 0.9294 | 1 | 172681031 | 41.1 |
| rs1323292 | A | G | 0.261754 | 0.025293 | 4.23E-25 | A | G | 0.00970278 | 0.0221 | 0.6609 | 1 | 192541021 | 107.0 |
| rs1378938 | C | T | -0.117783 | 0.0204027 | 7.79E-09 | C | T | 0.0144032 | 0.0186 | 0.4398 | 15 | 75096443 | 33.3 |
| rs182429 | G | A | -0.150143 | 0.0186583 | 8.49E-16 | G | A | -0.0437968 | 0.0173 | 0.0114101 | 6 | 159469574 | 64.7 |
| rs1893592 | C | A | -0.123864 | 0.0208749 | 2.96E-09 | C | A | -0.0489997 | 0.0197 | 0.01275 | 21 | 43855067 | 35.2 |
| rs1980422 | T | C | -0.172271 | 0.0215795 | 1.43E-15 | T | C | -0.0640053 | 0.0194 | 0.000957503 | 2 | 204610396 | 63.7 |
| rs2030519 | A | G | 0.27826 | 0.0188629 | 3E-49 | A | G | 0.0129952 | 0.0167 | 0.436 | 3 | 188119901 | 217.6 |
| rs2499714 | T | C | 0.188966 | 0.0305353 | 6.08E-10 | T | C | 0.0255995 | 0.0299 | 0.3918 | 6 | 34072215 | 38.2 |
| rs4445406 | C | T | -0.136049 | 0.0197343 | 5.42E-12 | C | T | -0.0495042 | 0.0178 | 0.00547898 | 1 | 2539400 | 47.5 |
| rs55743914 | T | C | 0.187309 | 0.021237 | 1.14E-18 | T | C | -0.0151035 | 0.0203 | 0.4571 | 6 | 128293562 | 77.7 |
| rs61579022 | A | G | 0.107957 | 0.0188338 | 9.92E-09 | A | G | -0.0116982 | 0.0171 | 0.4945 | 3 | 119123278 | 32.8 |
| rs6498114 | T | G | -0.131028 | 0.0211512 | 5.83E-10 | T | G | -0.0802994 | 0.0205 | 8.69701E-05 | 16 | 10964118 | 38.3 |
| rs6715106 | G | A | -0.237369 | 0.0412053 | 8.38E-09 | G | A | -0.0116024 | 0.0383 | 0.760801 | 2 | 191913034 | 33.1 |
| rs7104791 | C | T | -0.14842 | 0.0221049 | 1.89E-11 | C | T | -0.0297044 | 0.0205 | 0.1471 | 11 | 111196858 | 45.1 |
| rs744254 | A | G | 0.116421 | 0.0210172 | 3.04E-08 | A | G | 0.0146029 | 0.0188 | 0.4399 | 10 | 6392848 | 30.6 |
| rs76830965 | A | C | 0.307485 | 0.0283999 | 2.57E-27 | A | C | 0.0784969 | 0.0287 | 0.00614002 | 3 | 159637678 | 117.2 |
| rs79758729 | G | A | 0.162969 | 0.0290899 | 2.12E-08 | G | A | 0.0172988 | 0.0277 | 0.5309 | 7 | 37418454 | 31.3 |
| SNP | Effects on Celiac disease | | | | | Effects on Ulcerative colitis | | | | | Chr | Position | F  statistic |
|  | EA | OA | Beta | SE | p-val | EA | OA | Beta | SE | p-val |  |  |  |
| rs1018326 | C | T | 0.151862 | 0.0185859 | 3.06267E-16 | C | T | -0.0204985 | 0.0214 | 0.3372 | 2 | 182007800 | 66.7 |
| rs1050976 | T | C | -0.111043 | 0.0184718 | 1.83802E-09 | T | C | -0.00780034 | 0.0214 | 0.715201 | 6 | 408079 | 36.1 |
| rs10790269 | T | C | 0.157239 | 0.0239751 | 5.43751E-11 | T | C | 0.0202045 | 0.0271 | 0.4564 | 11 | 118729456 | 43.0 |
| rs10947460 | A | G | -0.12545 | 0.0227727 | 3.61302E-08 | A | G | -0.0196008 | 0.0253 | 0.4398 | 6 | 33828234 | 30.3 |
| rs11801183 | T | C | -0.138343 | 0.0245229 | 1.68702E-08 | T | C | 0.014997 | 0.0274 | 0.5841 | 1 | 172862984 | 31.8 |
| rs11851414 | C | T | 0.120446 | 0.0220512 | 4.705E-08 | C | T | 0.0608972 | 0.0254 | 0.0167101 | 14 | 69259502 | 29.8 |
| rs11875687 | C | T | 0.159565 | 0.0250579 | 1.91699E-10 | C | T | 0.0511986 | 0.0289 | 0.0763994 | 18 | 12843137 | 40.5 |
| rs12068671 | C | T | -0.156654 | 0.0244153 | 1.39701E-10 | C | T | 0.0222967 | 0.027 | 0.4102 | 1 | 172681031 | 41.1 |
| rs1250552 | G | A | -0.154784 | 0.0185777 | 7.96893E-17 | G | A | -0.0463968 | 0.0214 | 0.0303201 | 10 | 81058027 | 69.4 |
| rs1323292 | A | G | 0.261754 | 0.025293 | 4.23448E-25 | A | G | 0.00380276 | 0.028 | 0.8913 | 1 | 192541021 | 1071 |
| rs1378938 | C | T | -0.117783 | 0.0204027 | 7.79094E-09 | C | T | 0.0199986 | 0.0235 | 0.3955 | 15 | 75096443 | 33.3 |
| rs182429 | G | A | -0.150143 | 0.0186583 | 8.48594E-16 | G | A | 0.00359646 | 0.0219 | 0.8698 | 6 | 159469574 | 64.7 |
| rs1893592 | C | A | -0.123864 | 0.0208749 | 2.96299E-09 | C | A | -0.0565977 | 0.0244 | 0.0204 | 21 | 43855067 | 35.2 |
| rs1980422 | T | C | -0.172271 | 0.0215795 | 1.42692E-15 | T | C | -0.0827948 | 0.0244 | 0.000679094 | 2 | 204610396 | 63.7 |
| rs2030519 | A | G | 0.27826 | 0.0188629 | 2.99985E-49 | A | G | -0.0151035 | 0.0211 | 0.4742 | 3 | 188119901 | 217.6 |
| rs2097282 | T | C | -0.183987 | 0.0197345 | 1.13006E-20 | T | C | 0.0204005 | 0.0231 | 0.3773 | 3 | 46378025 | 86.9 |
| rs2499714 | T | C | 0.188966 | 0.0305353 | 6.07603E-10 | T | C | 0.0136958 | 0.0376 | 0.7165 | 6 | 34072215 | 38.2 |
| rs3184504 | C | T | -0.175633 | 0.0186829 | 5.41876E-21 | C | T | -0.0690035 | 0.0214 | 0.00124 | 12 | 111884608 | 88.3 |
| rs4821124 | C | T | 0.151003 | 0.0230512 | 5.724E-11 | C | T | 0.0998977 | 0.0262 | 0.0001356 | 22 | 21979289 | 42.9 |
| rs55743914 | T | C | 0.187309 | 0.021237 | 1.14499E-18 | T | C | 0.00169856 | 0.0255 | 0.9481 | 6 | 128293562 | 77.7 |
| rs61579022 | A | G | 0.107957 | 0.0188338 | 9.91996E-09 | A | G | -0.0309954 | 0.0216 | 0.1507 | 3 | 119123278 | 32.8 |
| rs61907765 | T | C | 0.161268 | 0.0221632 | 3.42926E-13 | T | C | 0.0768036 | 0.0255 | 0.00257502 | 11 | 128391937 | 52.9 |
| rs6498114 | T | G | -0.131028 | 0.0211512 | 5.83405E-10 | T | G | -0.0789999 | 0.0257 | 0.00209098 | 16 | 10964118 | 38.3 |
| rs6715106 | G | A | -0.237369 | 0.0412053 | 8.37896E-09 | G | A | -0.0142973 | 0.0482 | 0.7659 | 2 | 191913034 | 33.1 |
| rs7104791 | C | T | -0.14842 | 0.0221049 | 1.88886E-11 | C | T | -0.036004 | 0.0258 | 0.1625 | 11 | 111196858 | 45.1 |
| rs744254 | A | G | 0.116421 | 0.0210172 | 3.03599E-08 | A | G | -0.0143018 | 0.0237 | 0.545301 | 10 | 6392848 | 30.6 |
| rs76830965 | A | C | 0.307485 | 0.0283999 | 2.56507E-27 | A | C | 0.109903 | 0.0354 | 0.00190401 | 3 | 159637678 | 117.2 |
| rs79758729 | G | A | 0.162969 | 0.0290899 | 2.11602E-08 | G | A | 0.0657019 | 0.0342 | 0.0547205 | 7 | 37418454 | 31.3 |
| rs990171 | C | A | -0.178146 | 0.0215135 | 1.2249E-16 | C | A | -0.087003 | 0.0245 | 0.000389 | 2 | 103086770 | 68.5 |
| SNP | Effects on Celiac disease | | | | | Effects on Crohn's disease | | | | | Chr | Position | F  statistic |
|  | EA | OA | Beta | SE | p-val | EA | OA | Beta | SE | p-val |  |  |  |
| rs1018326 | C | T | 0.151862 | 0.0185859 | 3.06267E-16 | C | T | 0.00609856 | 0.0231 | 0.792301 | 2 | 182007800 | 66.7 |
| rs1050976 | T | C | -0.111043 | 0.0184718 | 1.83802E-09 | T | C | 0.0323994 | 0.0232 | 0.1629 | 6 | 408079 | 36.1 |
| rs10790269 | T | C | 0.157239 | 0.0239751 | 5.43751E-11 | T | C | 0.0402971 | 0.0295 | 0.1711 | 11 | 118729456 | 43.0 |
| rs10947460 | A | G | -0.12545 | 0.0227727 | 3.61302E-08 | A | G | -0.0108992 | 0.0277 | 0.6937 | 6 | 33828234 | 30.3 |
| rs11801183 | T | C | -0.138343 | 0.0245229 | 1.68702E-08 | T | C | -0.0492953 | 0.0301 | 0.1018 | 1 | 172862984 | 31.8 |
| rs11851414 | C | T | 0.120446 | 0.0220512 | 4.705E-08 | C | T | 0.0608015 | 0.0272 | 0.0254402 | 14 | 69259502 | 29.8 |
| rs12068671 | C | T | -0.156654 | 0.0244153 | 1.39701E-10 | C | T | -0.0198026 | 0.0294 | 0.4998 | 1 | 172681031 | 41.1 |
| rs13132308 | G | A | -0.34899 | 0.0269131 | 1.87499E-38 | G | A | -0.1067 | 0.0324 | 0.001007 | 4 | 123551114 | 168.1 |
| rs1323292 | A | G | 0.261754 | 0.025293 | 4.23448E-25 | A | G | 0.0214973 | 0.0301 | 0.4752 | 1 | 192541021 | 107.1 |
| rs1378938 | C | T | -0.117783 | 0.0204027 | 7.79094E-09 | C | T | 0.00660174 | 0.0252 | 0.7921 | 15 | 75096443 | 33.3 |
| rs17264332 | G | A | 0.250759 | 0.0220259 | 4.9831E-30 | G | A | 0.0391983 | 0.028 | 0.161 | 6 | 138005515 | 129.6 |
| rs1893592 | C | A | -0.123864 | 0.0208749 | 2.96299E-09 | C | A | -0.0251998 | 0.0276 | 0.3618 | 21 | 43855067 | 35.2 |
| rs1980422 | T | C | -0.172271 | 0.0215795 | 1.42692E-15 | T | C | -0.0385953 | 0.0264 | 0.1439 | 2 | 204610396 | 63.7 |
| rs2030519 | A | G | 0.27826 | 0.0188629 | 2.99985E-49 | A | G | 0.0456997 | 0.0229 | 0.0459801 | 3 | 188119901 | 217.6 |
| rs2499714 | T | C | 0.188966 | 0.0305353 | 6.07603E-10 | T | C | 0.0572967 | 0.0407 | 0.1595 | 6 | 34072215 | 38.2 |
| rs3184504 | C | T | -0.175633 | 0.0186829 | 5.41876E-21 | C | T | -0.0720974 | 0.0234 | 0.002048 | 12 | 111884608 | 88.3 |
| rs4445406 | C | T | -0.136049 | 0.0197343 | 5.42251E-12 | C | T | 0.0236986 | 0.0241 | 0.3267 | 1 | 2539400 | 47.5 |
| rs55743914 | T | C | 0.187309 | 0.021237 | 1.14499E-18 | T | C | -0.0385953 | 0.0276 | 0.1617 | 6 | 128293562 | 77.7 |
| rs61579022 | A | G | 0.107957 | 0.0188338 | 9.91996E-09 | A | G | -0.00110061 | 0.0233 | 0.9615 | 3 | 119123278 | 32.8 |
| rs61907765 | T | C | 0.161268 | 0.0221632 | 3.42926E-13 | T | C | 0.0858015 | 0.0275 | 0.00178398 | 11 | 128391937 | 52.9 |
| rs6498114 | T | G | -0.131028 | 0.0211512 | 5.83405E-10 | T | G | -0.0868005 | 0.0279 | 0.00188001 | 16 | 10964118 | 38.3 |
| rs6715106 | G | A | -0.237369 | 0.0412053 | 8.37896E-09 | G | A | -0.0528012 | 0.0523 | 0.3123 | 2 | 191913034 | 33.1 |
| rs7104791 | C | T | -0.14842 | 0.0221049 | 1.88886E-11 | C | T | -0.0358014 | 0.0278 | 0.1982 | 11 | 111196858 | 45.1 |
| rs744254 | A | G | 0.116421 | 0.0210172 | 3.03599E-08 | A | G | 0.0606999 | 0.0257 | 0.0180901 | 10 | 6392848 | 30.6 |
| rs76830965 | A | C | 0.307485 | 0.0283999 | 2.56507E-27 | A | C | 0.0125015 | 0.0403 | 0.7568 | 3 | 159637678 | 117.2 |
| rs79758729 | G | A | 0.162969 | 0.0290899 | 2.11602E-08 | G | A | -0.0477039 | 0.0386 | 0.2159 | 7 | 37418454 | 31.3 |

Abbreviation: EA, Effect Allele; OA, Other Allele; EAF, effect allele frequency; SE, standard error; SNP, single nucleotide polymorphism; Chr, Chromosome

**Table S2. Characteristics of the SNPs related to Celiac Disease and nflammatory Bowel Disease、Ulcerative colitis and Crohn's disease**

| SNP | Effects on Celiac disease | | | | | Effects on Inflammatory Bowel Disease | | | | | Chr | Position | F  statistic |
| --- | --- | --- | --- | --- | --- | --- | --- | --- | --- | --- | --- | --- | --- |
|  | EA | OA | Beta | SE | p-val | EA | OA | Beta | SE | p-val |  |  |  |
| rs1018326 | C | T | 0.151862 | 0.025768 | 3.78199E-09 | C | T | -0.0057037 | 0.017 | 0.736801 | 2 | 182007800 | 34.7 |
| rs11221335 | C | T | 0.217528 | 0.0329665 | 4.15528E-11 | C | T | 0.0862008 | 0.0199 | 0.00001492 | 11 | 128385906 | 43.5 |
| rs13098911 | T | C | 0.278389 | 0.0417266 | 2.52813E-11 | T | C | -0.00709511 | 0.0305 | 0.8146 | 3 | 46235201 | 44.5 |
| rs13151961 | G | A | -0.323931 | 0.0375492 | 6.30667E-18 | G | A | -0.118796 | 0.0236 | 4.60903E-07 | 4 | 123115502 | 74.4 |
| rs1738074 | C | T | -0.142367 | 0.0257295 | 3.14398E-08 | C | T | -0.040998 | 0.0172 | 0.0171199 | 6 | 159465977 | 30.6 |
| rs17810546 | G | A | 0.323532 | 0.0373431 | 4.56352E-18 | G | A | 0.0698003 | 0.0262 | 0.00768599 | 3 | 159665050 | 75.1 |
| rs2327832 | G | A | 0.231905 | 0.0301354 | 1.40994E-14 | G | A | 0.107596 | 0.0201 | 8.95695E-08 | 6 | 137973068 | 59.2 |
| rs2816316 | A | C | 0.254376 | 0.035932 | 1.4481E-12 | A | C | 0.0117013 | 0.0221 | 0.5947 | 1 | 192536813 | 50.1 |
| SNP | Effects on Celiac disease | | | | | Effects on Ulcerative colitis | | | | | Chr | Position | F  statistic |
|  | EA | OA | Beta | SE | p-val | EA | OA | Beta | SE | p-val |  |  |  |
| rs11221335 | C | T | 0.217528 | 0.0329665 | 4.16E-11 | C | T | 0.0812968 | 0.0251 | 0.00120401 | 11 | 128385906 | 43.5 |
| rs13098911 | T | C | 0.278389 | 0.0417266 | 2.53E-11 | T | C | -0.016597 | 0.0386 | 0.667599 | 3 | 46235201 | 44.5 |
| rs13151961 | G | A | -0.323931 | 0.0375492 | 6.31E-18 | G | A | -0.133901 | 0.0298 | 6.87907E-06 | 4 | 123115502 | 74.4 |
| rs1738074 | C | T | -0.142367 | 0.0257295 | 3.14E-08 | C | T | 0.00790113 | 0.0218 | 0.715801 | 6 | 159465977 | 30.6 |
| rs17810546 | G | A | 0.323532 | 0.0373431 | 4.56E-18 | G | A | 0.111803 | 0.0323 | 0.000544102 | 3 | 159665050 | 75.1 |
| rs2816316 | A | C | 0.254376 | 0.035932 | 1.45E-12 | A | C | 0.00740253 | 0.0279 | 0.7897 | 1 | 192536813 | 50.1 |
| rs653178 | T | C | -0.192272 | 0.0256108 | 6.03E-14 | T | C | -0.0611948 | 0.0211 | 0.00374999 | 12 | 112007756 | 56.3 |
| rs917997 | C | T | -0.231905 | 0.0297142 | 5.97E-15 | C | T | -0.087003 | 0.0245 | 0.000382904 | 2 | 103070568 | 60.9 |
| SNP | Effects on Celiac disease | | | | | Effects on Crohn's disease | | | | | Chr | Position | F  statistic |
|  | EA | OA | Beta | SE | p-val | EA | OA | Beta | SE | p-val |  |  |  |
| rs1018326 | C | T | 0.151862 | 0.025768 | 3.78199E-09 | C | T | 0.00609856 | 0.0231 | 0.792301 | 2 | 182007800 | 34.7 |
| rs11221335 | C | T | 0.217528 | 0.0329665 | 4.15528E-11 | C | T | 0.0885034 | 0.027 | 0.001036 | 11 | 128385906 | 43.5 |
| rs13098911 | T | C | 0.278389 | 0.0417266 | 2.52813E-11 | T | C | 0.0133998 | 0.0411 | 0.744501 | 3 | 46235201 | 44.5 |
| rs13151961 | G | A | -0.323931 | 0.0375492 | 6.30667E-18 | G | A | -0.110799 | 0.0323 | 0.000603698 | 4 | 123115502 | 74.4 |
| rs1464510 | A | C | 0.257738 | 0.0256539 | 9.49292E-24 | A | C | 0.0459958 | 0.023 | 0.0454004 | 3 | 188112554 | 100.9 |
| rs1738074 | C | T | -0.142367 | 0.0257295 | 3.14398E-08 | C | T | -0.105504 | 0.0233 | 6.05801E-06 | 6 | 159465977 | 30.6 |
| rs2327832 | G | A | 0.231905 | 0.0301354 | 1.40994E-14 | G | A | 0.034105 | 0.0279 | 0.2213 | 6 | 137973068 | 59.2 |
| rs2816316 | A | C | 0.254376 | 0.035932 | 1.4481E-12 | A | C | 0.0222995 | 0.03 | 0.4573 | 1 | 192536813 | 50.1 |
| rs653178 | T | C | -0.192272 | 0.0256108 | 6.02837E-14 | T | C | -0.0668024 | 0.0234 | 0.00429705 | 12 | 112007756 | 56.3 |

Abbreviation: EA, Effect Allele; OA, Other Allele; EAF, effect allele frequency; SE, standard error; SNP, single nucleotide polymorphism; Chr, Chromosome

**Table S3. Characteristics of the SNPs related to Inflammatory Bowel Disease、Ulcerative colitis and Crohn's disease with Celiac disease**

| SNP | Effects on Inflammatory Bowel Disease | | | | | | | | | | | Effects on Celiac disease | | | | | Chr | Position | F  statistic |
| --- | --- | --- | --- | --- | --- | --- | --- | --- | --- | --- | --- | --- | --- | --- | --- | --- | --- | --- | --- |
|  | EA | | | OA | | | Beta | | | SE | p-val | EA | OA | Beta | SE | p-val |  |  |  |
| rs10761659 | G | | | A | | | 0.161896 | | | 0.0172 | 4.07E-21 | G | A | -0.0207825 | 0.0187661 | 0.2681 | 10 | 64445564 | 88.5 |
| rs10800314 | A | | | C | | | -0.143097 | | | 0.0179 | 1.17E-15 | A | C | -0.0158733 | 0.0191352 | 0.4068 | 1 | 161472789 | 63.9 |
| rs11236797 | A | | | C | | | 0.155704 | | | 0.017 | 4.75E-20 | A | C | 0.0440169 | 0.018348 | 0.0164399 | 11 | 76299649 | 83.8 |
| rs11677953 | A | | | G | | | 0.0975985 | | | 0.0171 | 1.05E-08 | A | G | 0.0314987 | 0.0186764 | 0.0916896 | 2 | 219121663 | 32.5 |
| rs12764283 | A | | | G | | | 0.126597 | | | 0.0179 | 1.57E-12 | A | G | 0.0256677 | 0.0192058 | 0.1814 | 10 | 35530460 | 50.0 |
| rs12936409 | T | | | C | | | 0.145701 | | | 0.0168 | 3.87E-18 | T | C | -0.00702462 | 0.0184371 | 0.7032 | 17 | 38043649 | 75.2 |
| rs1551399 | C | | | A | | | 0.101302 | | | 0.0173 | 5.01E-09 | C | A | 0.037598 | 0.0188701 | 0.0463202 | 8 | 126539965 | 34.2 |
| rs1736161 | A | | | G | | | -0.123298 | | | 0.0174 | 1.34E-12 | A | G | -0.0425944 | 0.0186738 | 0.0225502 | 21 | 16833222 | 50.2 |
| rs17800987 | G | | | A | | | 0.2017 | | | 0.0305 | 3.71E-11 | G | A | 0.0497421 | 0.0325441 | 0.1264 | 5 | 150323428 | 43.7 |
| rs1873625 | A | | | C | | | 0.177301 | | | 0.0179 | 3.71E-23 | A | C | 0.0582689 | 0.0200395 | 0.00364099 | 3 | 49666964 | 98.1 |
| rs2076756 | G | | | A | | | 0.187595 | | | 0.0186 | 5.59E-24 | G | A | 0.0478373 | 0.0214579 | 0.0257899 | 16 | 50756881 | 101.7 |
| rs2193041 | G | | | A | | | 0.133703 | | | 0.0172 | 6.91E-15 | G | A | 0.0178399 | 0.0189661 | 0.3469 | 12 | 68502110 | 60.4 |
| rs35260072 | C | | | A | | | 0.142197 | | | 0.017 | 7.07E-17 | C | A | 0.0525924 | 0.0187493 | 0.00503095 | 5 | 131630852 | 69.9 |
| rs4246905 | C | | | T | | | 0.163001 | | | 0.0197 | 1.42E-16 | C | T | 0.057841 | 0.0202487 | 0.00428302 | 9 | 117553249 | 68.4 |
| rs4730272 | G | | | A | | | -0.134102 | | | 0.0178 | 4.5E-14 | G | A | -0.0158733 | 0.0185398 | 0.3919 | 7 | 107478227 | 56.7 |
| rs6062496 | A | | | G | | | 0.164997 | | | 0.018 | 5.48E-20 | A | G | 0.00531409 | 0.0187711 | 0.7771 | 20 | 62329099 | 84.0 |
| rs6584283 | C | | | T | | | -0.180303 | | | 0.0169 | 1.7E-26 | C | T | 0.00380724 | 0.0182446 | 0.8347 | 10 | 101290301 | 113.8 |
| rs6826501 | T | | | C | | | -0.0927953 | | | 0.0169 | 4.12E-08 | T | C | -0.00995033 | 0.0186264 | 0.5932 | 4 | 36076676 | 30.1 |
| rs6880778 | G | | | A | | | 0.187801 | | | 0.0173 | 2.14E-27 | G | A | 0.0305623 | 0.0188964 | 0.1058 | 5 | 40399096 | 117.8 |
| rs6911490 | C | | | T | | | -0.142801 | | | 0.0208 | 6.82E-12 | C | T | -0.0421012 | 0.0227109 | 0.0637705 | 6 | 106522027 | 47.1 |
| rs744166 | G | | | A | | | -0.120703 | | | 0.0172 | 2.16E-12 | G | A | -0.00803217 | 0.0186857 | 0.6673 | 17 | 40514201 | 49.2 |
| SNP | Effects on Ulcerative colitis | | | | | | | | | | | Effects on Celiac disease | | | | | Chr | Position | F  statistic |
|  | EA | OA | | | | Beta | | | SE | | p-val | EA | OA | Beta | SE | p-val |  |  |  |
| rs11209026 | A | G | | | | -0.561698 | | | 0.0517 | | 1.58E-27 | A | G | 0.00796817 | 0.0368486 | 0.8288 | 1 | 67705958 | 118.0 |
| rs114152040 | A | G | | | | 0.339603 | | | 0.0623 | | 4.95E-08 | A | G | 0.0610951 | 0.0528861 | 0.248 | 5 | 40444986 | 29.7 |
| rs1359946 | A | G | | | | 0.158302 | | | 0.0269 | | 3.84E-09 | A | G | 0.0246926 | 0.0237311 | 0.2981 | 13 | 27536972 | 34.6 |
| rs1801274 | G | A | | | | -0.182896 | | | 0.0217 | | 3.78E-17 | G | A | 0.00974735 | 0.0184257 | 0.5968 | 1 | 161479745 | 71.0 |
| rs3024493 | A | C | | | | 0.236297 | | | 0.0276 | | 1.09E-17 | A | C | -0.0456252 | 0.0256738 | 0.0755492 | 1 | 206943968 | 73.2 |
| rs3829111 | A | G | | | | 0.156303 | | | 0.0214 | | 2.89E-13 | A | G | 0.0487902 | 0.0186434 | 0.00886993 | 9 | 139269483 | 53.3 |
| rs4676410 | A | G | | | | 0.207802 | | | 0.0284 | | 2.46E-13 | A | G | 0.0198026 | 0.0228384 | 0.3859 | 2 | 241563739 | 53.5 |
| rs483905 | A | G | | | | 0.128903 | | | 0.0228 | | 1.57E-08 | A | G | 0.001998 | 0.0184794 | 0.9139 | 11 | 96023427 | 31.9 |
| rs56167332 | A | C | | | | 0.151596 | | | 0.0231 | | 5.3E-11 | A | C | 0.00895974 | 0.0192954 | 0.642401 | 5 | 158827769 | 43.1 |
| rs6017342 | C | A | | | | 0.191306 | | | 0.024 | | 1.38E-15 | C | A | -0.0188218 | 0.0190286 | 0.3226 | 20 | 43065028 | 63.5 |
| rs6062496 | A | G | | | | 0.158498 | | | 0.0224 | | 1.47E-12 | A | G | 0.00531409 | 0.0187711 | 0.7771 | 20 | 62329099 | 50.1 |
| rs7752873 | T | C | | | | 0.182297 | | | 0.0303 | | 1.83E-09 | T | C | 0.0334348 | 0.0268475 | 0.213 | 6 | 106579332 | 36.1 |
| rs9977672 | A | G | | | | -0.245006 | | | 0.0261 | | 6.21E-21 | A | G | -0.0129839 | 0.0210781 | 0.537901 | 21 | 40463283 | 88.1 |
| SNP | Effects on Crohn's disease | | | | | | | | | | | Effects on Celiac Disease | | | | | Chr | Position | F  statistic |
|  | EA | | OA | | Beta | | | SE | | | p-val | EA | OA | Beta | SE | p-val |  |  |  |
| rs10761659 | G | | A | | 0.212006 | | | 0.0237 | | | 3.41979E-19 | G | A | -0.0207825 | 0.0187661 | 0.2681 | 10 | 64445564 | 80.0 |
| rs11236797 | A | | C | | 0.181104 | | | 0.0231 | | | 4.854E-15 | A | C | 0.0440169 | 0.018348 | 0.0164399 | 11 | 76299649 | 61.4 |
| rs1456896 | T | | C | | 0.139301 | | | 0.0251 | | | 2.89701E-08 | T | C | 0.0283995 | 0.0199751 | 0.1551 | 7 | 50304461 | 30.8 |
| rs1873625 | A | | C | | 0.180704 | | | 0.0243 | | | 1.09396E-13 | A | C | 0.0582689 | 0.0200395 | 0.00364099 | 3 | 49666964 | 55.2 |
| rs2076756 | G | | A | | 0.399806 | | | 0.0242 | | | 3.24489E-61 | G | A | 0.0478373 | 0.0214579 | 0.0257899 | 16 | 50756881 | 272.9 |
| rs2188962 | T | | C | | 0.212398 | | | 0.0228 | | | 1.35988E-20 | T | C | 0.0582689 | 0.0189431 | 0.00209802 | 5 | 131770805 | 86.7 |
| rs281379 | A | | G | | 0.139797 | | | 0.0238 | | | 4.26403E-09 | A | G | 0.0517144 | 0.0184121 | 0.00497405 | 19 | 49214274 | 34.5 |
| rs28701841 | A | | G | | 0.224303 | | | 0.0373 | | | 1.85102E-09 | A | G | 0.0344014 | 0.0293717 | 0.2415 | 6 | 106530330 | 36.1 |
| rs3024505 | A | | G | | 0.177903 | | | 0.0302 | | | 3.90499E-09 | A | G | -0.0459392 | 0.0256649 | 0.0734598 | 1 | 206939904 | 34.7 |
| rs3091315 | G | | A | | -0.179501 | | | 0.0263 | | | 9.52138E-12 | G | A | 0.0237165 | 0.0209583 | 0.2578 | 17 | 32593665 | 46.5 |
| rs4902642 | A | | G | | -0.129198 | | | 0.0236 | | | 4.339E-08 | A | G | -0.0119714 | 0.0188422 | 0.5252 | 14 | 69210199 | 29.9 |
| rs697693 | A | | G | | 0.172296 | | | 0.0281 | | | 8.35507E-10 | A | G | 0.00299551 | 0.0252593 | 0.9056 | 1 | 7886424 | 37.5 |
| rs744166 | G | | A | | -0.129299 | | | 0.0233 | | | 2.92301E-08 | G | A | -0.00803217 | 0.0186857 | 0.6673 | 17 | 40514201 | 30.7 |
| rs907092 | A | | G | | 0.130396 | | | 0.0228 | | | 1.011E-08 | A | G | -0.00410843 | 0.0187764 | 0.9537 | 17 | 37922259 | 32.7 |
| rs921720 | G | | A | | 0.162895 | | | 0.0237 | | | 6.39588E-12 | G | A | 0.0403013 | 0.0189282 | 0.3129 | 8 | 126534671 | 47.2 |

Abbreviation: EA, Effect Allele; OA, Other Allele; EAF, effect allele frequency; SE, standard error; SNP, single nucleotide polymorphism; Chr, Chromosome

**Table S4. Characteristics of the SNPs related to Inflammatory Bowel Disease、Ulcerative colitis and Crohn's disease with Celiac disease**

| SNP | Effects on Inflammatory Bowel Disease | | | | | | | | | | | Effects on Celiac disease | | | | | Chr | Position | F  statistic |
| --- | --- | --- | --- | --- | --- | --- | --- | --- | --- | --- | --- | --- | --- | --- | --- | --- | --- | --- | --- |
|  | EA | | | OA | | | Beta | | | SE | p-val | EA | OA | Beta | SE | p-val |  |  |  |
| rs1042058 | C | | | T | | | 0.0709591 | | | 0.0111998 | 2.09498E-10 | C | T | 0.0332466 | 0.0188554 | 0.0778592 | 10 | 30728101 | 40.1 |
| rs1050152 | T | | | C | | | 0.144966 | | | 0.0111013 | 4.13809E-39 | T | C | 0.0535408 | 0.0188909 | 0.00459399 | 5 | 131676320 | 170.5 |
| rs1062158 | T | | | C | | | 0.0806579 | | | 0.0113986 | 1.21199E-12 | T | C | 0.078935 | 0.0192672 | 4.18803E-05 | 5 | 141523000 | 50.1 |
| rs10758669 | A | | | C | | | -0.161343 | | | 0.0114999 | 7.88134E-45 | A | C | -0.0227395 | 0.0196259 | 0.2466 | 9 | 4981602 | 196.8 |
| rs10761659 | G | | | A | | | 0.156537 | | | 0.0111 | 8.46837E-45 | G | A | -0.0207825 | 0.0187661 | 0.2681 | 10 | 64445564 | 198.9 |
| rs10781499 | A | | | G | | | 0.176471 | | | 0.0111996 | 4.38026E-56 | A | G | 0.0487902 | 0.0186729 | 0.00897801 | 9 | 139266405 | 248.3 |
| rs10800309 | G | | | A | | | -0.139762 | | | 0.0116006 | 2.56389E-33 | G | A | -0.0158733 | 0.0192375 | 0.4093 | 1 | 161472158 | 145.2 |
| rs11564126 | G | | | A | | | 0.341786 | | | 0.0342001 | 1.66802E-23 | G | A | 0.227932 | 0.0644919 | 0.000408903 | 12 | 40820001 | 99.9 |
| rs11597483 | G | | | A | | | 0.112497 | | | 0.0113999 | 5.23721E-23 | G | A | 0.0217615 | 0.0190334 | 0.2529 | 10 | 35285117 | 97.4 |
| rs11742570 | C | | | T | | | 0.183442 | | | 0.0113 | 3.46338E-59 | C | T | 0.0283995 | 0.0188592 | 0.1321 | 5 | 40410584 | 263.5 |
| rs11879191 | A | | | G | | | -0.121151 | | | 0.0138001 | 2.0361E-18 | A | G | -0.0326265 | 0.0231705 | 0.1591 | 19 | 10512911 | 77.1 |
| rs12142199 | A | | | G | | | -0.0977231 | | | 0.0150002 | 7.58578E-11 | A | G | 0.0196926 | 0.0231666 | 0.3953 | 1 | 1249187 | 42.4 |
| rs12722515 | A | | | C | | | -0.090253 | | | 0.0155 | 5.82197E-09 | A | C | -0.0308717 | 0.0258624 | 0.2326 | 10 | 6081230 | 33.9 |
| rs1292053 | G | | | A | | | 0.0697789 | | | 0.0109 | 1.76701E-10 | G | A | 0.0129162 | 0.0181876 | 0.4776 | 17 | 57963537 | 40.9 |
| rs12942547 | G | | | A | | | -0.107957 | | | 0.0111994 | 5.50554E-22 | G | A | -0.00934352 | 0.0187469 | 0.6182 | 17 | 40527544 | 92.9 |
| rs12946510 | T | | | C | | | 0.142367 | | | 0.0109997 | 4.1011E-38 | T | C | -0.00420884 | 0.0186337 | 0.8213 | 17 | 37912377 | 167.5 |
| rs13009506 | T | | | G | | | 0.136278 | | | 0.0110001 | 3.47216E-35 | T | G | -0.0188218 | 0.0184249 | 0.307 | 2 | 234175706 | 153.5 |
| rs1456896 | T | | | C | | | 0.0889262 | | | 0.0118998 | 9.75439E-14 | T | C | 0.0283995 | 0.0199751 | 0.1551 | 7 | 50304461 | 55.8 |
| rs1517352 | C | | | A | | | 0.0749391 | | | 0.0112999 | 3.28171E-11 | C | A | 0.0161294 | 0.0189346 | 0.3943 | 2 | 191931464 | 43.9 |
| rs1558744 | A | | | G | | | 0.110647 | | | 0.0111989 | 2.63027E-23 | A | G | 0.0168571 | 0.0191695 | 0.3792 | 12 | 68504592 | 97.6 |
| rs17085007 | C | | | T | | | 0.104805 | | | 0.0143999 | 3.75405E-13 | C | T | 0.0227395 | 0.0235621 | 0.3345 | 13 | 27531267 | 52.9 |
| rs17119 | A | | | G | | | 0.0934903 | | | 0.0141008 | 3.07468E-11 | A | G | -0.0363319 | 0.0228481 | 0.1118 | 6 | 14719496 | 43.9 |
| rs17293632 | T | | | C | | | 0.0998453 | | | 0.0127007 | 5.91562E-15 | T | C | 0.08158 | 0.0216168 | 0.000160702 | 15 | 67442596 | 61.8 |
| rs1872691 | A | | | G | | | -0.12579 | | | 0.0146 | 5.58727E-18 | A | G | -0.0222456 | 0.0241311 | 0.3566 | 16 | 50350210 | 74.2 |
| rs2024092 | A | | | G | | | 0.108854 | | | 0.0136 | 1.22999E-15 | A | G | 0.0861777 | 0.0225092 | 0.000128899 | 19 | 1124031 | 64.1 |
| rs2155219 | T | | | G | | | 0.138892 | | | 0.0111002 | 4.23643E-36 | T | G | 0.0369752 | 0.0185107 | 0.0457699 | 11 | 76299194 | 156.6 |
| rs2382817 | C | | | A | | | -0.076961 | | | 0.0110984 | 3.70425E-12 | C | A | -0.0295588 | 0.0190322 | 0.1204 | 2 | 219151218 | 48.1 |
| rs2413583 | T | | | C | | | -0.181762 | | | 0.0151999 | 4.40352E-33 | T | C | 0.0237165 | 0.0241962 | 0.327 | 22 | 39659773 | 142.9 |
| rs2488389 | A | | | G | | | 0.0907544 | | | 0.0132009 | 5.21555E-12 | A | G | 0.0324672 | 0.0225706 | 0.1503 | 1 | 197631141 | 47.3 |
| rs259964 | G | | | A | | | -0.0788112 | | | 0.0111015 | 1.01298E-12 | G | A | -0.0497421 | 0.0186429 | 0.00762693 | 20 | 57824309 | 50.4 |
| rs2823286 | A | | | G | | | -0.140988 | | | 0.0124002 | 9.27684E-30 | A | G | -0.0347985 | 0.0204126 | 0.0882409 | 21 | 16817938 | 129.3 |
| rs2836878 | A | | | G | | | -0.177095 | | | 0.0128999 | 7.2711E-43 | A | G | -0.0170444 | 0.0209102 | 0.415 | 21 | 40465534 | 188.5 |
| rs3197999 | A | | | G | | | 0.170586 | | | 0.0118012 | 1.00693E-47 | A | G | 0.064851 | 0.0201374 | 0.00128 | 3 | 49721532 | 208.9 |
| rs3764147 | G | | | A | | | 0.0923346 | | | 0.0128002 | 5.63119E-13 | G | A | 0.0188218 | 0.0224923 | 0.4027 | 13 | 44457925 | 52.0 |
| rs4072037 | T | | | C | | | 0.0685928 | | | 0.0111988 | 6.86894E-10 | T | C | -0.00895974 | 0.0194062 | 0.6443 | 1 | 155162067 | 37.5 |
| rs4246905 | C | | | T | | | 0.148268 | | | 0.0125 | 2.80414E-32 | C | T | 0.057841 | 0.0202487 | 0.00428302 | 9 | 117553249 | 140.7 |
| rs4409764 | G | | | T | | | -0.171429 | | | 0.0110006 | 1.02896E-54 | G | T | 0.00300451 | 0.0186772 | 0.8722 | 10 | 101284237 | 242.8 |
| rs4656958 | G | | | A | | | 0.0691357 | | | 0.0119 | 6.79908E-09 | G | A | 0.0296348 | 0.0200147 | 0.1387 | 1 | 160856964 | 33.8 |
| rs559928 | C | | | T | | | 0.0948603 | | | 0.0143999 | 4.18697E-11 | C | T | 0.0415514 | 0.0240034 | 0.0834392 | 11 | 64150370 | 43.4 |
| rs5743289 | T | | | C | | | 0.242946 | | | 0.0137998 | 2.90202E-69 | T | C | 0.0525924 | 0.0243727 | 0.03094 | 16 | 50756774 | 309.9 |
| rs6017342 | C | | | A | | | 0.119572 | | | 0.0125 | 1.42692E-21 | C | A | -0.0188218 | 0.0190286 | 0.3226 | 20 | 43065028 | 91.5 |
| rs6062504 | G | | | A | | | 0.1207 | | | 0.0120002 | 1.09194E-23 | G | A | 0.00350614 | 0.0197507 | 0.8591 | 20 | 62348907 | 101.2 |
| rs6426833 | A | | | G | | | 0.122218 | | | 0.0110001 | 2.01883E-28 | A | G | -0.001998 | 0.0190341 | 0.9164 | 1 | 20171860 | 123.4 |
| rs6724516 | A | | | G | | | 0.079735 | | | 0.0126007 | 2.229E-10 | A | G | 0.0372866 | 0.0207657 | 0.0725605 | 2 | 241586810 | 40.0 |
| rs6908425 | C | | | T | | | 0.0980539 | | | 0.0135 | 3.36977E-13 | C | T | 0.0349021 | 0.0224362 | 0.1198 | 6 | 20728731 | 52.8 |
| rs6911490 | C | | | T | | | -0.119559 | | | 0.0137991 | 3.80365E-18 | C | T | -0.0421012 | 0.0227109 | 0.0637705 | 6 | 106522027 | 75.1 |
| rs7097656 | C | | | T | | | 0.105916 | | | 0.0139 | 2.0488E-14 | C | T | 0.0343844 | 0.0232578 | 0.1393 | 10 | 82250831 | 58.1 |
| rs7240004 | G | | | A | | | -0.0667236 | | | 0.0115991 | 9.80596E-09 | G | A | -0.0694573 | 0.0190575 | 0.0002678 | 18 | 46395022 | 33.1 |
| rs762422 | A | | | G | | | -0.117658 | | | 0.0112001 | 6.52079E-26 | A | G | -0.0188218 | 0.0192303 | 0.3277 | 21 | 45615638 | 110.4 |
| rs7749278 | C | | | T | | | 0.0881757 | | | 0.0110001 | 8.45279E-16 | C | T | -0.0296348 | 0.0184748 | 0.1087 | 6 | 167435325 | 64.6 |
| rs8005161 | T | | | C | | | 0.144966 | | | 0.0190003 | 2.34909E-14 | T | C | 0.00299551 | 0.0351055 | 0.932 | 14 | 88472595 | 58.2 |
| rs913678 | C | | | T | | | -0.064851 | | | 0.0118001 | 4.59198E-08 | C | T | -0.00340579 | 0.0195071 | 0.8614 | 20 | 48955424 | 30.2 |
| rs9170 | A | | | G | | | 0.108854 | | | 0.0163992 | 3.07468E-11 | A | G | 0.0834216 | 0.027934 | 0.00282299 | 2 | 43450138 | 44.1 |
| rs921720 | G | | | A | | | 0.0882849 | | | 0.0113002 | 6.70656E-15 | G | A | 0.0403013 | 0.0189282 | 0.0332399 | 8 | 126534671 | 61.0 |
| rs941823 | C | | | T | | | 0.0804511 | | | 0.0128002 | 3.83698E-10 | C | T | 0.0323166 | 0.0214548 | 0.132 | 13 | 41013977 | 39.5 |
| SNP | Effects on Ulcerative colitis | | | | | | | | | | | Effects on Celiac disease | | | | | Chr | Position | F  statistic |
|  | EA | OA | | | | Beta | | | SE | | p-val | EA | OA | Beta | SE | p-val |  |  |  |
| rs1026916 | G | A | | | | 0.0887219 | | | 0.0188099 | | 2.79383E-11 | G | A | -0.0119286 | 0.0187341 | 0.5243 | 17 | 40529835 | 22.2 |
| rs1050152 | T | C | | | | 0.0806579 | | | 0.0179099 | | 9.46891E-11 | T | C | 0.0535408 | 0.0188909 | 0.00459399 | 5 | 131676320 | 20.2 |
| rs10758669 | A | C | | | | -0.155293 | | | 0.0183899 | | 6.96466E-29 | A | C | -0.0227395 | 0.0196259 | 0.2466 | 9 | 4981602 | 71.3 |
| rs10761659 | G | A | | | | 0.118333 | | | 0.01784 | | 4.94652E-17 | G | A | -0.0207825 | 0.0187661 | 0.2681 | 10 | 64445564 | 43.9 |
| rs10781499 | A | G | | | | 0.149282 | | | 0.0179194 | | 3.90122E-28 | A | G | 0.0487902 | 0.0186729 | 0.00897801 | 9 | 139266405 | 69.4 |
| rs11041476 | A | G | | | | 0.0778865 | | | 0.0189903 | | 6.98602E-10 | A | G | -0.027988 | 0.0200194 | 0.1621 | 11 | 1875067 | 16.8 |
| rs11150589 | C | T | | | | -0.0861777 | | | 0.0177412 | | 6.03698E-10 | C | T | 0.0225524 | 0.018531 | 0.2236 | 16 | 30482494 | 23.5 |
| rs11168249 | C | T | | | | 0.0760347 | | | 0.0177399 | | 7.78108E-09 | C | T | 0.0129162 | 0.0185056 | 0.4852 | 12 | 48208368 | 18.3 |
| rs11209026 | A | G | | | | -0.520203 | | | 0.0411802 | | 3.20184E-62 | A | G | 0.00796817 | 0.0368486 | 0.8288 | 1 | 67705958 | 159.5 |
| rs11597483 | G | A | | | | 0.0639133 | | | 0.0184 | | 3.67502E-09 | G | A | 0.0217615 | 0.0190334 | 0.2529 | 10 | 35285117 | 12.1 |
| rs11739663 | C | T | | | | -0.0692429 | | | 0.021279 | | 1.81201E-08 | C | T | -0.0339705 | 0.0217429 | 0.1182 | 5 | 594083 | 10.6 |
| rs12103 | C | T | | | | -0.105261 | | | 0.0226005 | | 2.73401E-10 | C | T | 0.0228593 | 0.0236715 | 0.3342 | 1 | 1247494 | 21.6 |
| rs12720356 | C | A | | | | 0.126633 | | | 0.0308199 | | 3.96798E-10 | C | A | -0.071496 | 0.0332255 | 0.0314101 | 19 | 10469975 | 16.8 |
| rs12946510 | T | C | | | | 0.151003 | | | 0.0177702 | | 3.38532E-26 | T | C | -0.00420884 | 0.0186337 | 0.8213 | 17 | 37912377 | 72.2 |
| rs1422878 | T | C | | | | 0.142367 | | | 0.01855 | | 5.40381E-19 | T | C | 0.0119286 | 0.0191617 | 0.5336 | 5 | 158839217 | 58.9 |
| rs17085007 | C | T | | | | 0.153579 | | | 0.0227301 | | 2.78805E-19 | C | T | 0.0227395 | 0.0235621 | 0.3345 | 13 | 27531267 | 45.6 |
| rs17229285 | T | C | | | | -0.111157 | | | 0.0177299 | | 1.72504E-13 | T | C | -0.00697561 | 0.017915 | 0.697 | 2 | 199523122 | 39.3 |
| rs1728785 | C | A | | | | 0.0731085 | | | 0.02134 | | 3.70997E-08 | C | A | -0.0246926 | 0.021853 | 0.2585 | 16 | 68591230 | 11.7 |
| rs17539176 | G | A | | | | 0.162969 | | | 0.0349101 | | 1.53993E-11 | G | A | -0.0368715 | 0.0384193 | 0.3372 | 6 | 111777219 | 21.7 |
| rs1801274 | G | A | | | | -0.175783 | | | 0.0179791 | | 2.1208E-38 | G | A | 0.00974735 | 0.0184257 | 0.5968 | 1 | 161479745 | 95.5 |
| rs2111485 | G | A | | | | -0.08158 | | | 0.0180579 | | 1.93099E-08 | G | A | -0.00598207 | 0.0184453 | 0.7457 | 2 | 163110536 | 20.4 |
| rs2155219 | T | G | | | | 0.100373 | | | 0.0178602 | | 1.78608E-15 | T | G | 0.0369752 | 0.0185107 | 0.0457699 | 11 | 76299194 | 31.5 |
| rs2310173 | G | T | | | | -0.0907544 | | | 0.0177115 | | 3.89493E-13 | G | T | -0.00399202 | 0.019861 | 0.8407 | 2 | 102663628 | 26.2 |
| rs2413583 | T | C | | | | -0.145026 | | | 0.02443 | | 1.15904E-13 | T | C | 0.0237165 | 0.0241962 | 0.327 | 22 | 39659773 | 35.2 |
| rs2651244 | A | G | | | | -0.0713886 | | | 0.01817 | | 2.29097E-08 | A | G | 0.0129162 | 0.0186035 | 0.4875 | 1 | 70995562 | 15.4 |
| rs2836878 | A | G | | | | -0.236102 | | | 0.02072 | | 4.61849E-48 | A | G | -0.0170444 | 0.0209102 | 0.415 | 21 | 40465534 | 129.8 |
| rs3024505 | A | G | | | | 0.244514 | | | 0.0232807 | | 6.94065E-41 | A | G | -0.0459392 | 0.0256649 | 0.0734598 | 1 | 206939904 | 110.3 |
| rs3742704 | C | A | | | | 0.130151 | | | 0.03071 | | 1.55299E-08 | C | A | 0.0009995 | 0.0271192 | 0.9706 | 14 | 88477882 | 17.9 |
| rs3749171 | T | C | | | | 0.154436 | | | 0.0228707 | | 3.06902E-21 | T | C | 0.0392207 | 0.0244578 | 0.1088 | 2 | 241569692 | 45.5 |
| rs4722672 | T | C | | | | -0.0870947 | | | 0.0225499 | | 2.06101E-08 | T | C | -0.00697561 | 0.0239334 | 0.770699 | 7 | 27231762 | 14.9 |
| rs4728142 | A | G | | | | 0.0989399 | | | 0.017842 | | 4.37119E-14 | A | G | 0.00697561 | 0.0190127 | 0.7137 | 7 | 128573967 | 30.7 |
| rs561722 | T | C | | | | -0.113729 | | | 0.01914 | | 5.14754E-17 | T | C | -0.0147076 | 0.0196339 | 0.453799 | 11 | 114386830 | 35.3 |
| rs5763634 | C | T | | | | -0.10075 | | | 0.0200892 | | 2.96825E-11 | C | T | 0.0179603 | 0.0212093 | 0.3971 | 22 | 30350532 | 25.1 |
| rs6017342 | C | A | | | | 0.205918 | | | 0.0181899 | | 1.43087E-43 | C | A | -0.0188218 | 0.0190286 | 0.3226 | 20 | 43065028 | 128.1 |
| rs6062504 | G | A | | | | 0.0781778 | | | 0.01939 | | 7.49376E-11 | G | A | 0.00350614 | 0.0197507 | 0.8591 | 20 | 62348907 | 16.2 |
| rs6088765 | G | T | | | | 0.0760347 | | | 0.0180502 | | 2.20602E-08 | G | T | 0.0468836 | 0.0185789 | 0.0116201 | 20 | 33799280 | 17.7 |
| rs6584283 | C | T | | | | -0.164667 | | | 0.0177887 | | 1.91382E-34 | C | T | 0.00380724 | 0.0182446 | 0.8347 | 10 | 101290301 | 85.6 |
| rs6889364 | A | G | | | | 0.115113 | | | 0.0257108 | | 2.68782E-11 | A | G | 0.0256677 | 0.0276933 | 0.354 | 5 | 40347469 | 20.1 |
| rs7134599 | A | G | | | | 0.144966 | | | 0.0180807 | | 8.5055E-32 | A | G | 0.0178399 | 0.0192158 | 0.3532 | 12 | 68500075 | 64.2 |
| rs7184802 | A | G | | | | -0.0864297 | | | 0.0232198 | | 9.105E-09 | A | G | -0.0323166 | 0.0240535 | 0.1791 | 16 | 50355996 | 13.8 |
| rs7404095 | C | T | | | | 0.0675297 | | | 0.01797 | | 1.15401E-08 | C | T | -0.0266419 | 0.0189295 | 0.1593 | 16 | 23864590 | 14.1 |
| rs762422 | A | G | | | | -0.0870947 | | | 0.0181602 | | 4.75664E-15 | A | G | -0.0188218 | 0.0192303 | 0.3277 | 21 | 45615638 | 23.0 |
| rs941823 | C | T | | | | 0.0969515 | | | 0.02084 | | 2.95325E-11 | C | T | 0.0323166 | 0.0214548 | 0.132 | 13 | 41013977 | 21.6 |
| SNP | Effects on Crohn's disease | | | | | | | | | | | Effects on Celiac Disease | | | | | Chr | Position | F  statistic |
|  | EA | | OA | | Beta | | | SE | | | p-val | EA | OA | Beta | SE | p-val |  |  |  |
| rs10065637 | T | | C | | -0.116871 | | | 0.0204998 | | | 3.68E-12 | T | C | -0.0462534 | 0.0227974 | 0.0424698 | 5 | 55438851 | 32.5 |
| rs10127727 | A | | G | | 0.0953102 | | | 0.0194619 | | | 6.51E-09 | A | G | 0.0629748 | 0.0220097 | 0.00421998 | 1 | 173150964 | 23.9 |
| rs1042058 | C | | T | | 0.08534 | | | 0.01691 | | | 5.93E-11 | C | T | 0.0332466 | 0.0188554 | 0.0778592 | 10 | 30728101 | 25.4 |
| rs10758669 | A | | C | | -0.164667 | | | 0.0171001 | | | 1.44E-31 | A | C | -0.0227395 | 0.0196259 | 0.2466 | 9 | 4981602 | 92.7 |
| rs10761659 | G | | A | | 0.185125 | | | 0.0166999 | | | 6.37E-46 | G | A | -0.0207825 | 0.0187661 | 0.2681 | 10 | 64445564 | 122.8 |
| rs10781499 | A | | G | | 0.189794 | | | 0.0167295 | | | 3.48E-48 | A | G | 0.0487902 | 0.0186729 | 0.00897801 | 9 | 139266405 | 128.7 |
| rs10800309 | G | | A | | -0.0953102 | | | 0.017441 | | | 6.38E-12 | G | A | -0.0158733 | 0.0192375 | 0.4093 | 1 | 161472158 | 29.8 |
| rs11177083 | T | | C | | -0.0776373 | | | 0.0166301 | | | 1.14E-08 | T | C | -0.0113643 | 0.0186688 | 0.5427 | 12 | 68571545 | 21.7 |
| rs11741861 | G | | A | | 0.280657 | | | 0.0288799 | | | 2.94E-37 | G | A | 0.0497421 | 0.0340113 | 0.1436 | 5 | 150277909 | 94.4 |
| rs11742570 | C | | T | | 0.258123 | | | 0.01727 | | | 1.81E-82 | C | T | 0.0283995 | 0.0188592 | 0.1321 | 5 | 40410584 | 223.3 |
| rs11879191 | A | | G | | -0.133874 | | | 0.0209302 | | | 1.78E-17 | A | G | -0.0326265 | 0.0231705 | 0.1591 | 19 | 10512911 | 40.9 |
| rs12722515 | A | | C | | -0.118108 | | | 0.0234298 | | | 3.76E-10 | A | C | -0.0308717 | 0.0258624 | 0.2326 | 10 | 6081230 | 25.4 |
| rs1292053 | G | | A | | 0.0925792 | | | 0.0165198 | | | 8.85E-13 | G | A | 0.0129162 | 0.0181876 | 0.4776 | 17 | 57963537 | 31.4 |
| rs12942547 | G | | A | | -0.107697 | | | 0.01686 | | | 3.34E-17 | G | A | -0.00934352 | 0.0187469 | 0.6182 | 17 | 40527544 | 40.8 |
| rs12994997 | A | | G | | 0.209981 | | | 0.0167115 | | | 4.14E-70 | A | G | -0.0188218 | 0.0190325 | 0.3227 | 2 | 234173503 | 157.8 |
| rs13126505 | A | | G | | 0.158712 | | | 0.0322494 | | | 2.33E-10 | A | G | -0.0296348 | 0.036162 | 0.4125 | 4 | 102865304 | 24.2 |
| rs1456896 | T | | C | | 0.101037 | | | 0.0180305 | | | 7.27E-15 | T | C | 0.0283995 | 0.0199751 | 0.1551 | 7 | 50304461 | 31.4 |
| rs1517352 | C | | A | | 0.0897059 | | | 0.01704 | | | 1.93E-10 | C | A | 0.0161294 | 0.0189346 | 0.3943 | 2 | 191931464 | 27.7 |
| rs16967103 | C | | T | | 0.0843411 | | | 0.0205299 | | | 3.88E-09 | C | T | 0.0497421 | 0.0234598 | 0.0339797 | 15 | 38899190 | 16.8 |
| rs17391694 | T | | C | | -0.12613 | | | 0.0262201 | | | 2.96E-09 | T | C | -0.0541394 | 0.0282872 | 0.0556301 | 1 | 78623626 | 23.1 |
| rs194749 | C | | T | | 0.0843411 | | | 0.01959 | | | 2.7E-10 | C | T | 0.0129162 | 0.0227673 | 0.5705 | 14 | 69273905 | 18.5 |
| rs2155219 | T | | G | | 0.175306 | | | 0.0166013 | | | 9.56E-36 | T | G | 0.0369752 | 0.0185107 | 0.0457699 | 11 | 76299194 | 111.5 |
| rs2188962 | T | | C | | 0.202124 | | | 0.0167599 | | | 1.35E-52 | T | C | 0.0582689 | 0.0189431 | 0.00209802 | 5 | 131770805 | 145.4 |
| rs2284553 | G | | A | | 0.116759 | | | 0.01704 | | | 2.14E-16 | G | A | -0.0009995 | 0.0155957 | 0.9489 | 21 | 34776695 | 46.9 |
| rs2413583 | T | | C | | -0.221021 | | | 0.0228799 | | | 4.35E-31 | T | C | 0.0237165 | 0.0241962 | 0.327 | 22 | 39659773 | 93.3 |
| rs2488389 | A | | G | | 0.115113 | | | 0.0199097 | | | 8.45E-13 | A | G | 0.0324672 | 0.0225706 | 0.1503 | 1 | 197631141 | 33.4 |
| rs259964 | G | | A | | -0.0934903 | | | 0.016581 | | | 3.87E-11 | G | A | -0.0497421 | 0.0186429 | 0.00762693 | 20 | 57824309 | 31.7 |
| rs26528 | C | | T | | 0.125751 | | | 0.01667 | | | 9.65E-22 | C | T | -0.0265493 | 0.0187099 | 0.1559 | 16 | 28517709 | 56.9 |
| rs2790216 | A | | G | | -0.0737542 | | | 0.0204302 | | | 8.07E-09 | A | G | 0.0129162 | 0.0224751 | 0.565501 | 10 | 59997926 | 13.0 |
| rs2823286 | A | | G | | -0.168537 | | | 0.01846 | | | 4.63E-28 | A | G | -0.0347985 | 0.0204126 | 0.0882409 | 21 | 16817938 | 83.3 |
| rs2945412 | A | | G | | 0.128743 | | | 0.0168408 | | | 8.68E-17 | A | G | 0.0222456 | 0.018784 | 0.2363 | 17 | 25843643 | 58.4 |
| rs3024505 | A | | G | | 0.151003 | | | 0.0220815 | | | 2.48E-19 | A | G | -0.0459392 | 0.0256649 | 0.0734598 | 1 | 206939904 | 46.7 |
| rs3197999 | A | | G | | 0.150143 | | | 0.01786 | | | 5.31E-29 | A | G | 0.064851 | 0.0201374 | 0.00128 | 3 | 49721532 | 70.6 |
| rs3764147 | G | | A | | 0.1441 | | | 0.0191399 | | | 2.19E-21 | G | A | 0.0188218 | 0.0224923 | 0.4027 | 13 | 44457925 | 56.6 |
| rs4246905 | C | | T | | 0.135934 | | | 0.0185901 | | | 6.22E-26 | C | T | 0.057841 | 0.0202487 | 0.00428302 | 9 | 117553249 | 53.4 |
| rs4409764 | G | | T | | -0.167825 | | | 0.0165814 | | | 1.8E-41 | G | T | 0.00300451 | 0.0186772 | 0.8722 | 10 | 101284237 | 102.4 |
| rs529866 | T | | C | | -0.157707 | | | 0.02129 | | | 1.73E-16 | T | C | -0.0903624 | 0.0236245 | 0.000130801 | 16 | 11373320 | 54.8 |
| rs5743289 | T | | C | | 0.442761 | | | 0.0198297 | | | 5.61E-166 | T | C | 0.0525924 | 0.0243727 | 0.03094 | 16 | 50756774 | 498.5 |
| rs6062504 | G | | A | | 0.117771 | | | 0.0181199 | | | 2.45E-19 | G | A | 0.00350614 | 0.0197507 | 0.8591 | 20 | 62348907 | 42.2 |
| rs6500315 | G | | A | | 0.151288 | | | 0.02014 | | | 5.42E-23 | G | A | 0.03335 | 0.0219459 | 0.1286 | 16 | 50508101 | 56.4 |
| rs743479 | T | | C | | -0.101654 | | | 0.0168101 | | | 1.76E-20 | T | C | -0.0198026 | 0.0190473 | 0.2985 | 21 | 45611950 | 36.5 |
| rs7532161 | G | | A | | 0.139837 | | | 0.0167202 | | | 1.9E-23 | G | A | 0.0221434 | 0.0186219 | 0.2344 | 1 | 67642223 | 69.9 |
| rs8014798 | A | | C | | 0.1441 | | | 0.0287306 | | | 1.53E-12 | A | C | 0.00697561 | 0.0321061 | 0.828 | 14 | 88456559 | 25.1 |
| rs907092 | A | | G | | 0.143234 | | | 0.0164705 | | | 2.69E-25 | A | G | -0.00410843 | 0.0187764 | 0.8268 | 17 | 37922259 | 75.6 |
| rs921720 | G | | A | | 0.107363 | | | 0.0171498 | | | 8.3E-20 | G | A | 0.0403013 | 0.0189282 | 0.0332399 | 8 | 126534671 | 39.1 |
| rs9313808 | G | | A | | 0.130337 | | | 0.0227201 | | | 4.88E-17 | G | A | -0.0009995 | 0.0172146 | 0.9537 | 5 | 158820844 | 32.9 |
| rs9358372 | A | | G | | -0.101654 | | | 0.0170401 | | | 8.66E-14 | A | G | -0.0188218 | 0.018651 | 0.3129 | 6 | 20812588 | 35.5 |

Abbreviation: EA, Effect Allele; OA, Other Allele; EAF, effect allele frequency; SE, standard error; SNP, single nucleotide polymorphism; Chr, Chromosome

**Table S5 Removed pleiotropic SNPs associated with celiac disease and inflammatory bowel disease, ulcerative colitis and Crohn's disease**

| Exposure traits | Outcome traits | PhenoScanner database | MR-PRESSO outlier test | F-statistic < 10 | leave-one-out analysis |
| --- | --- | --- | --- | --- | --- |
| CeD | IBD | rs13003464, rs990171 ,rs17264332, rs3184504, rs11875687, rs4821124 | rs61907765, rs1250552, rs13195040, rs13195040, rs13132308, rs2097282 | NA | NA |
|  | UC | rs13003464, rs17264332 | rs13195040, rs13132308, rs4445406 | NA | NA |
|  | CD | rs13003464, rs990171, rs11875687, rs4821124 | rs13195040, rs1250552, rs182429, rs2097282 | NA | NA |

MR, Mendelian Randomization; MR-PRESSO, MR pleiotropy residual sum and outlier; CeD, Celiac Disease; IBD, Inflammatory Bowel Disease; UC, Ulcerative Colitis; CD, Crohn's Disease.

**Table S6 Removed pleiotropic SNPs associated with celiac disease and inflammatory bowel disease, ulcerative colitis and Crohn's disease**

| Exposure traits | Outcome traits | PhenoScanner database | MR-PRESSO outlier test | F-statistic < 10 | leave-one-out analysis |
| --- | --- | --- | --- | --- | --- |
| CeD | IBD | rs13003464, rs917997, rs653178 | rs1464510 | NA | NA |
|  | UC | rs13003464, rs2327832 | rs1464510, 、rs1018326 | NA | NA |
|  | CD | rs13003464, rs917997 | rs17810546 | NA | NA |

MR, Mendelian Randomization; MR-PRESSO, MR pleiotropy residual sum and outlier; CeD, Celiac Disease; IBD, Inflammatory Bowel Disease; UC, Ulcerative Colitis; CD, Crohn's Disease.

**Table S7** **Removed pleiotropic SNPs associated with inflammatory bowel disease, ulcerative colitis and Crohn's disease and celiac disease**

| Exposure traits | CeD | | | |
| --- | --- | --- | --- | --- |
|  | PhenoScanner database | MR-PRESSO outlier test | F-statistic < 10 | leave-one-out analysis |
| IBD | rs6927172 | rs11209026, rs3024493, rs7523335, rs254560, rs1003342, | NA | NA |
| UC | rs6933404 | rs4574921, rs7523335 | NA | NA |
| CD | rs114607072, rs80262450 | rs11209026 | NA | NA |

MR, Mendelian Randomization; MR-PRESSO, MR pleiotropy residual sum and outlier; CeD, Celiac Disease; IBD, Inflammatory Bowel Disease; UC, Ulcerative Colitis; CD, Crohn's Disease.

**Table S8 Removed pleiotropic SNPs associated with inflammatory bowel disease, ulcerative colitis and Crohn's disease and celiac disease**

| Exposure traits | CeD | | | |
| --- | --- | --- | --- | --- |
|  | PhenoScanner database | MR-PRESSO outlier test | F-statistic < 10 | leave-one-out analysis |
| IBD | rs7657746, rs6920220, rs6927022, rs1893217 | rs7554511, rs3024505, rs11209026, rs7608910, rs13019081, rs10516487, rs1847472, rs4743820, rs1250550, rs11230563, rs243323, rs727088, rs516246, rs2266959 | NA | NA |
| UC | rs1512973, rs6927022, rs6920220, rs1893217 | rs7523335, rs7554511, rs7608910, rs3197999, rs4743820, rs28374715, rs7240004 | rs6911490, rs483905 | NA |
| CD | rs917997, rs1893217 | rs6679677, rs11209026, rs7554511, rs7583409,rs7608910, rs4256159, rs4145717, rs212388, rs1819333, rs1847472, rs13204742, rs6651252, rs1250546, rs16967103, rs516246, rs2024092, rs2266959, rs714027 | rs7583409 | NA |

MR, Mendelian Randomization; MR-PRESSO, MR pleiotropy residual sum and outlier; CeD, Celiac Disease; IBD, Inflammatory Bowel Disease; UC, Ulcerative Colitis; CD, Crohn's Disease.

**Table S9 Heterogeneity and pleiotropy analysis of CeD with IBD, UC, and CD, using different analytical methods.**

| Exposure traits | Outcome traits | MR methods | Cochran Q statistic | Heterogeneity p-value | Pleiotropy p-value | MR-PRESSO global outlier test | |
| --- | --- | --- | --- | --- | --- | --- | --- |
|  |  |  |  |  |  | RSSOBs | p-value |
| CeD | IBD | Inverse variance weighted | 29.8703 | 0.0001 | 0.6949 | 38.8461 | 0.004 |
|  | UC | Inverse variance weighted | 19.9443 | 0.0056 | 0.3858 | 25.3865 | 0.016 |
|  | CD | Inverse variance weighted | 19.1306 | 0.0141 | 0.3637 | 22.888 | 0.05 |

MR, Mendelian Randomization; MR-PRESSO, MR pleiotropy residual sum and outlier; CeD, Celiac Disease; IBD, Inflammatory Bowel Disease; UC, Ulcerative Colitis; CD, Crohn's Disease.

**Table S10 Heterogeneity and pleiotropy analysis of IBD, UC, and CD with PSC, using different analytical methods.**

| Exposure traits | MR methods | CeD | | | | |
| --- | --- | --- | --- | --- | --- | --- |
|  |  | Cochran Q statistic | Heterogeneity p-value | Pleiotropy p-value | MR-PRESSO global outlier test | |
|  |  |  |  |  | RSSOBs | p-value |
| IBD | Inverse variance weighted | 120.0972 | 6.22E-07 | 0.1061 | 124.43 | <0.001 |
| UC | Inverse variance weighted | 63.5189 | 0.0176 | 0.1823 | 66.2503 | 0.014 |
| CD | Inverse variance weighted | 82.202 | 0.0008 | 0.1302 | 85.965 | 0.002 |

MR, Mendelian Randomization; MR-PRESSO, MR pleiotropy residual sum and outlie; IBD, Inflammatory Bowel Disease; UC, Ulcerative Colitis; CD, Crohn's Disease; CeD, Celiac Disease.


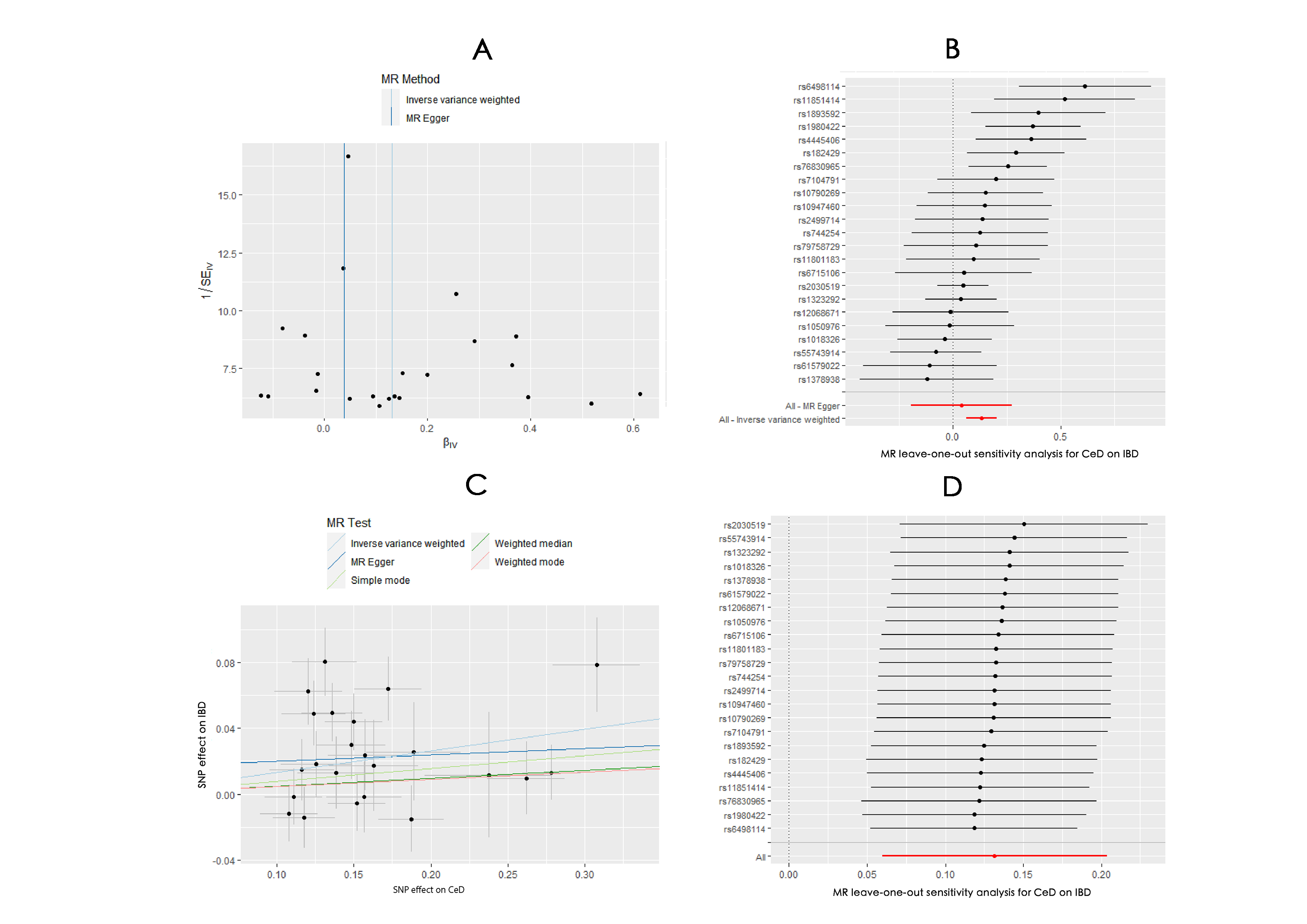


Figure S1 Funnel plot (A), forest plot (B), scatter plot (C), and leave-one-out analysis (D) of the causal effect of CeD on IBD risk.


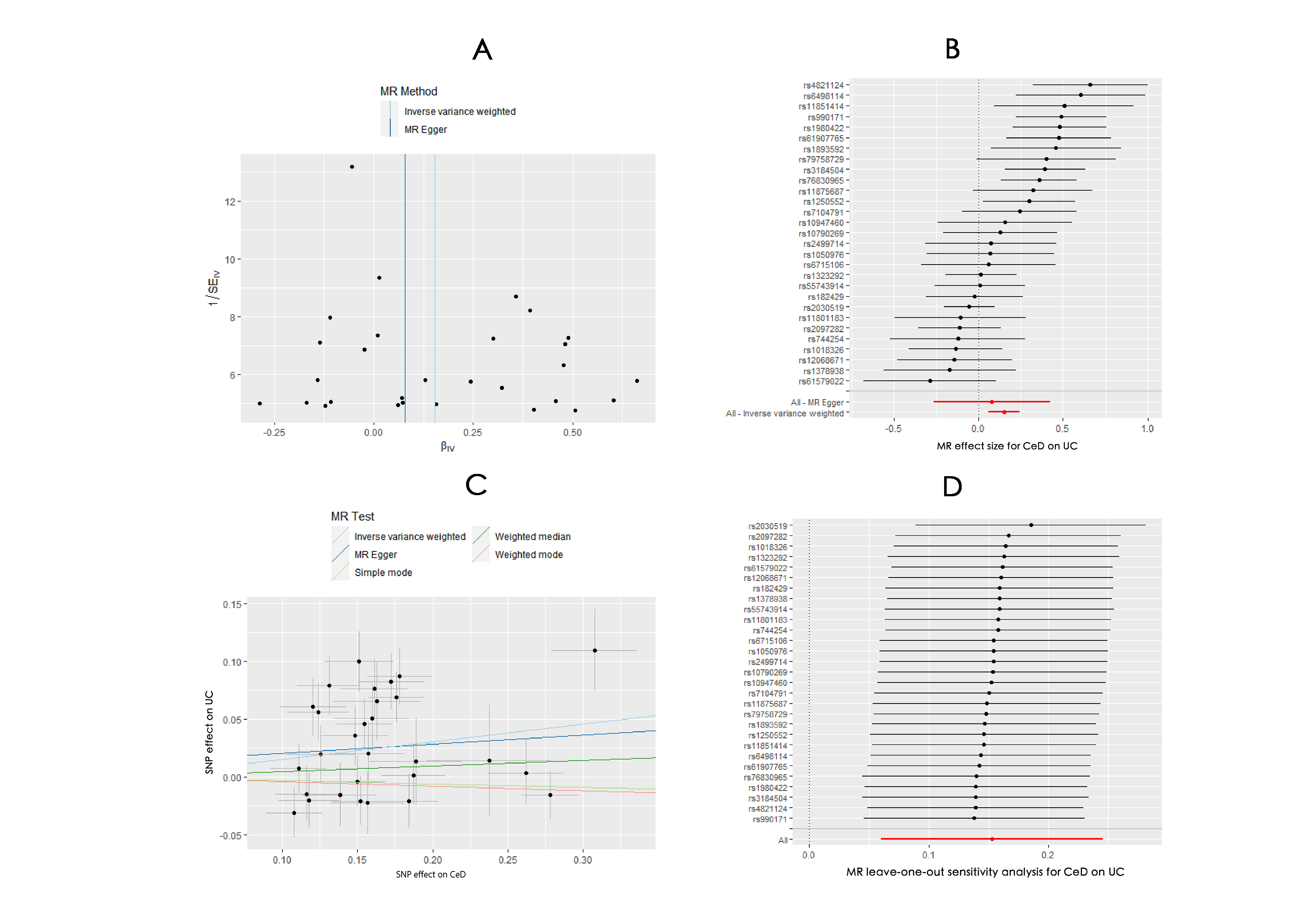


Figure S2 Funnel plot (A), forest plot (B), scatter plot (C), and leave-one-out analysis (D) of the causal effect of CeD on UC risk.


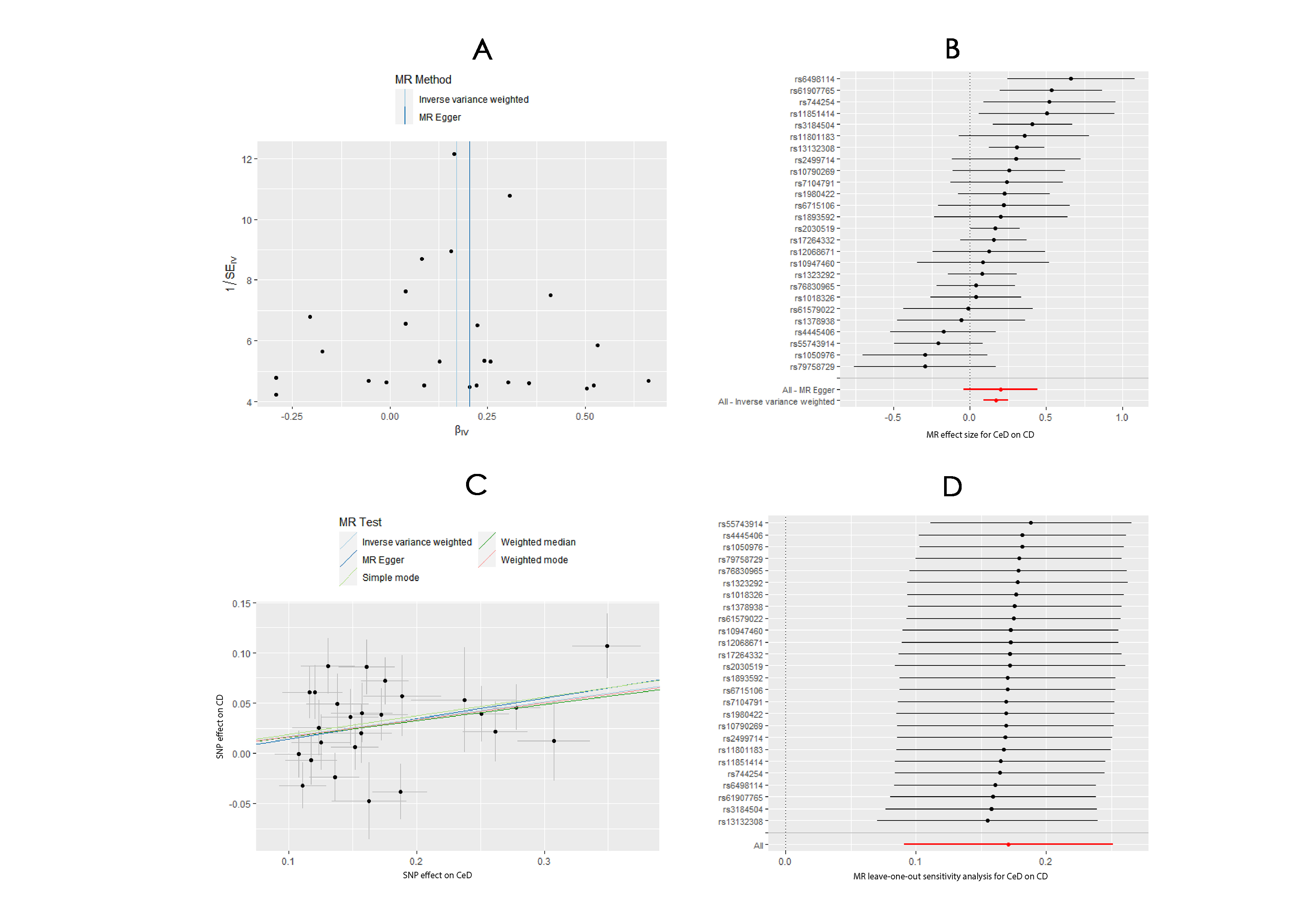


Figure S3 Funnel plot (A), forest plot (B), scatter plot (C), and leave-one-out analysis (D) of the causal effect of CeD on CD risk.


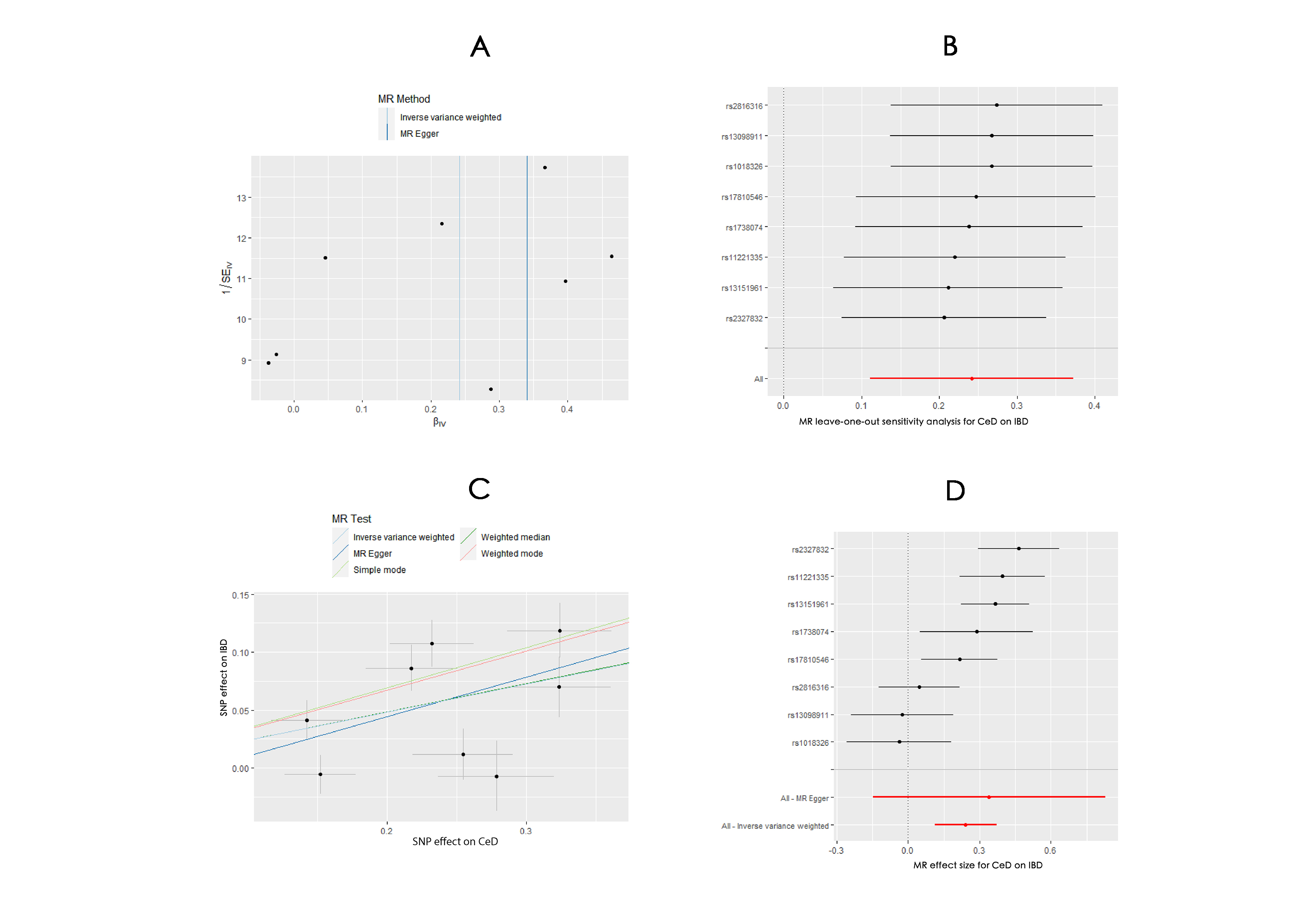


Figure S4 Funnel plot (A), forest plot (B), scatter plot (C), and leave-one-out analysis (D) of the causal effect of CeD on IBD risk.


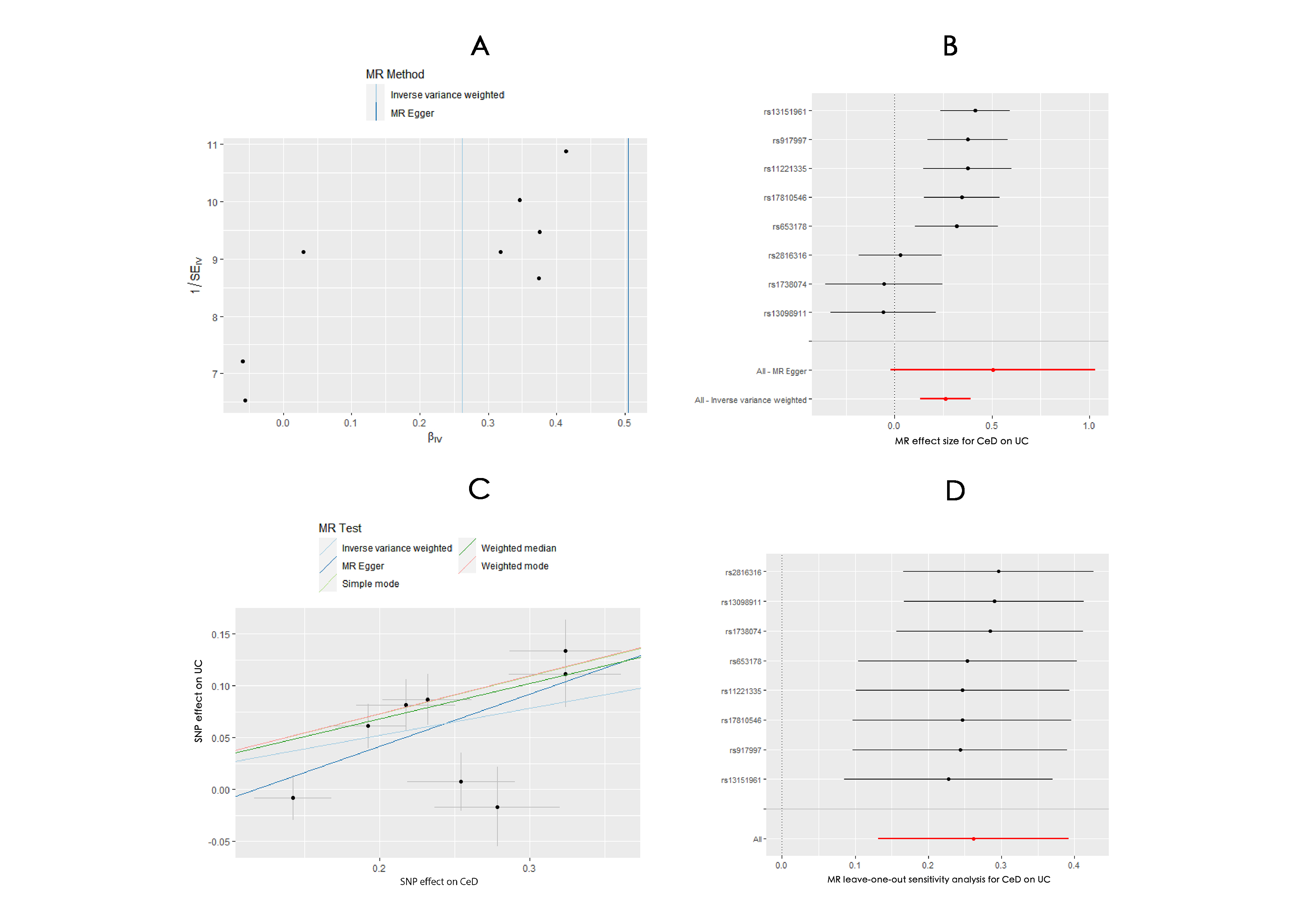


Figure S5 Funnel plot (A), forest plot (B), scatter plot (C), and leave-one-out analysis (D) of the causal effect of CeD on UC risk.


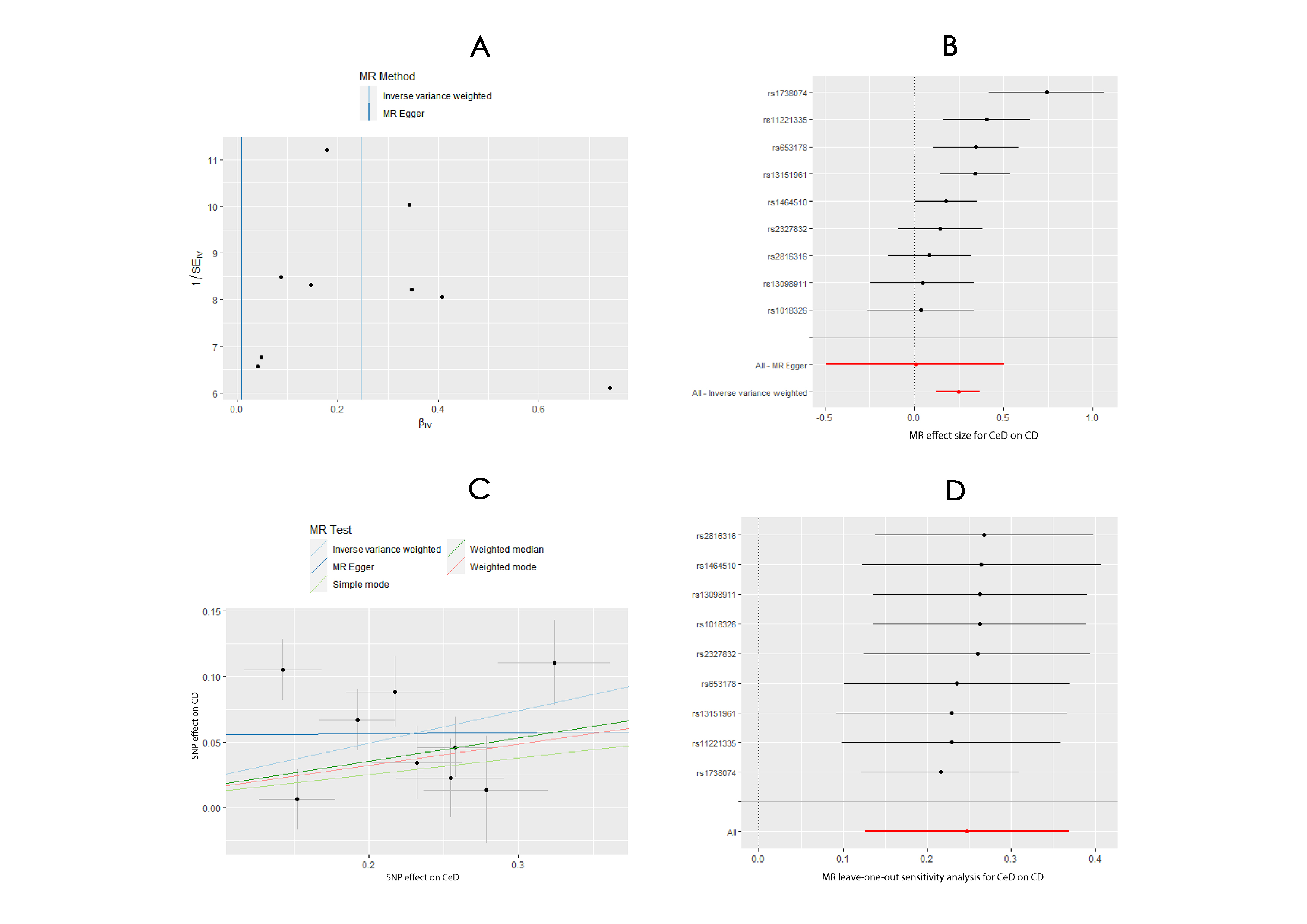


Figure S6 Funnel plot (A), forest plot (B), scatter plot (C), and leave-one-out analysis (D) of the causal effect of CeD on CD risk.


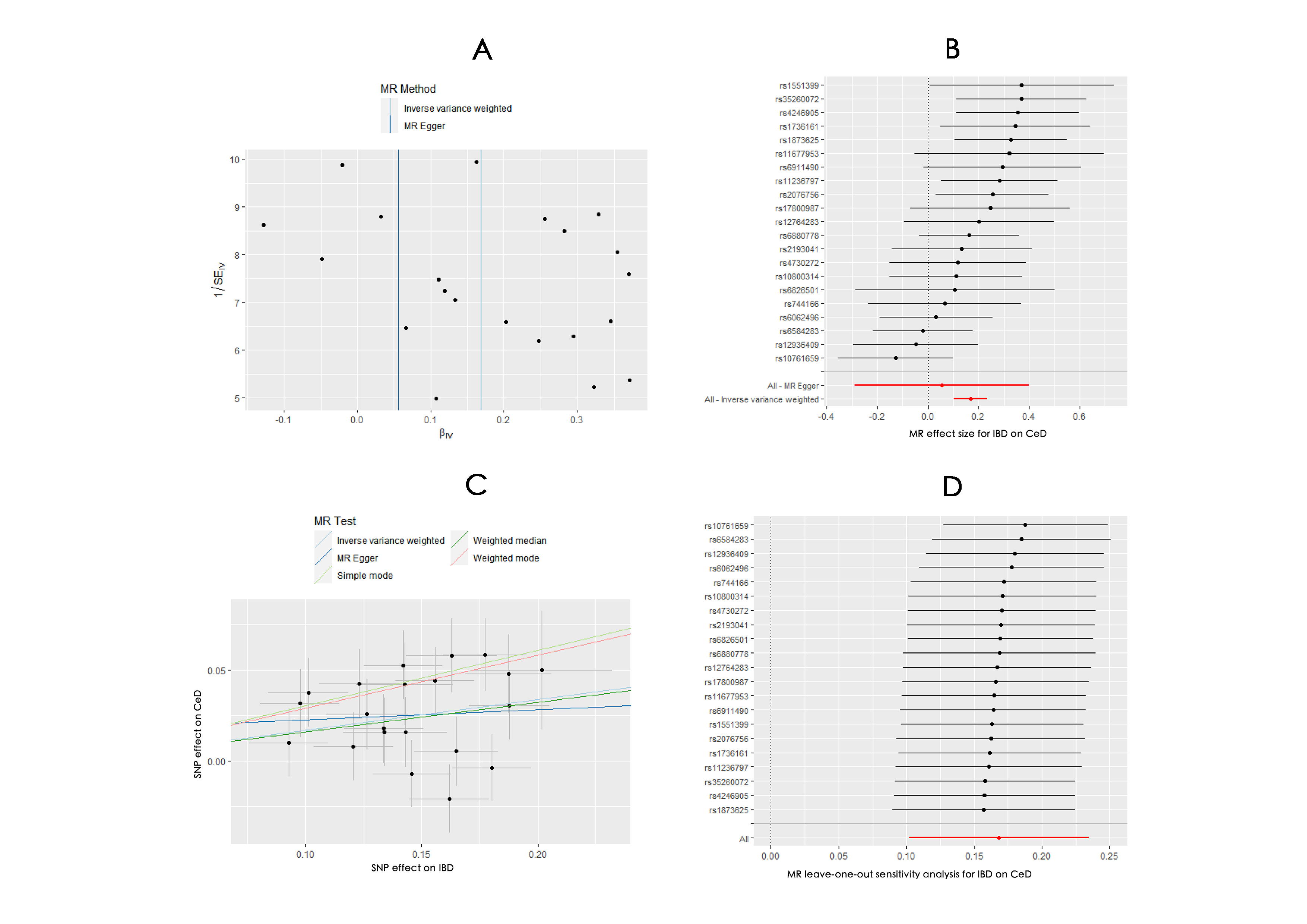


Figure S7 Funnel plot (A), forest plot (B), scatter plot (C), and leave-one-out analysis (D) of the causal effect of IBD on CeD risk.


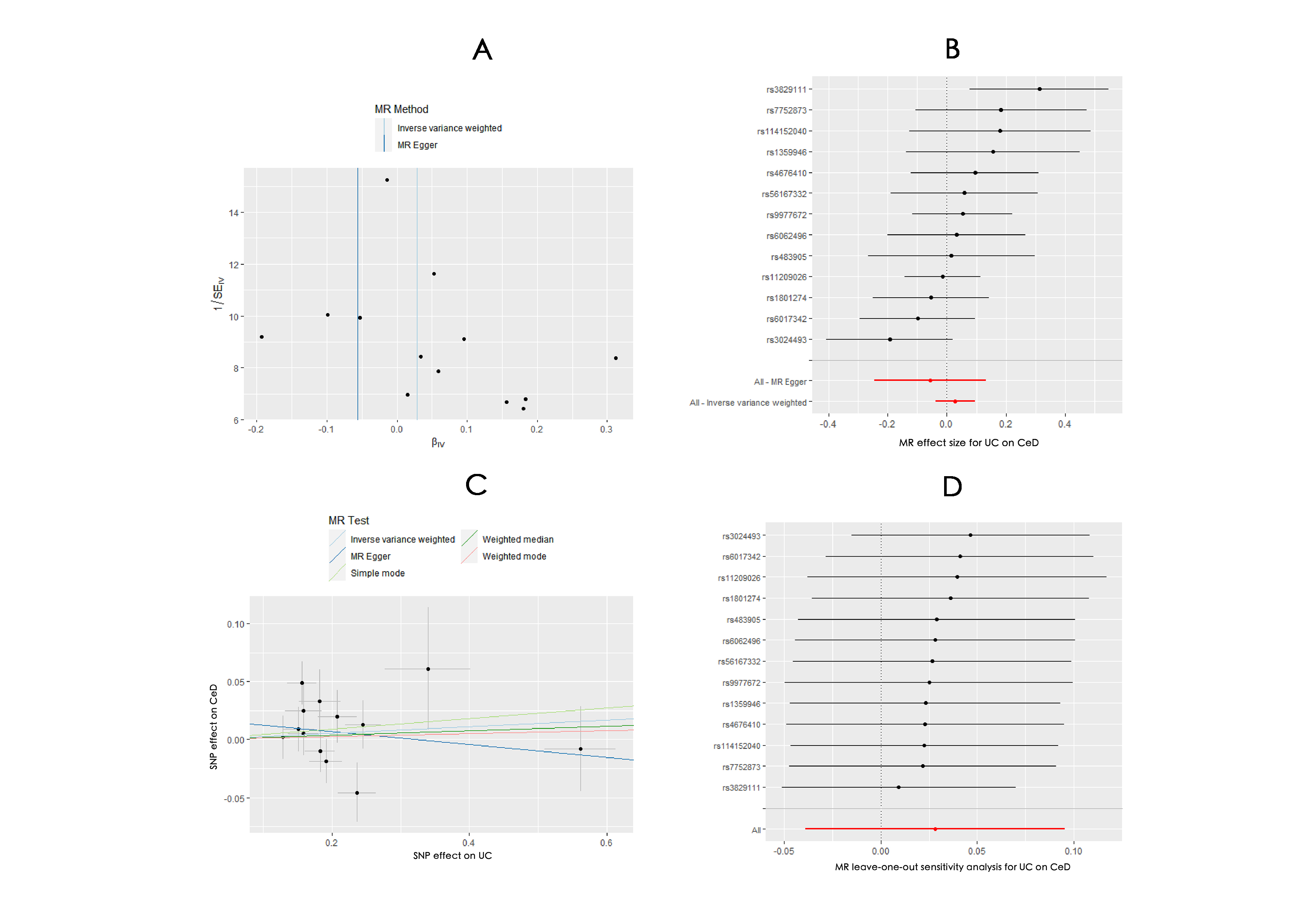


Figure S8 Funnel plot (A), forest plot (B), scatter plot (C), and leave-one-out analysis (D) of the causal effect of UC on CeD risk.


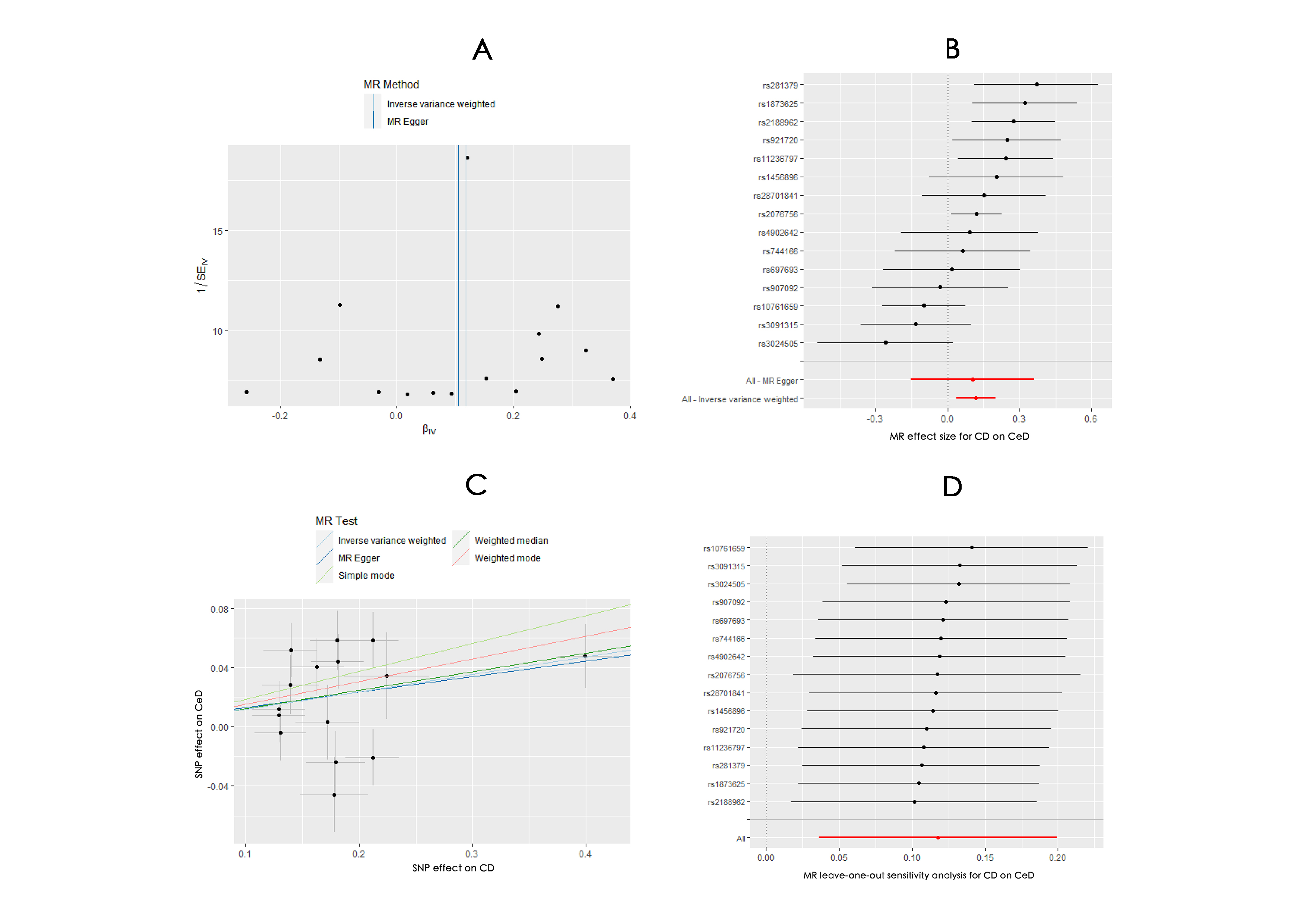


Figure S9 Funnel plot (A), forest plot (B), scatter plot (C), and leave-one-out analysis (D) of the causal effect of CD on CeD risk.


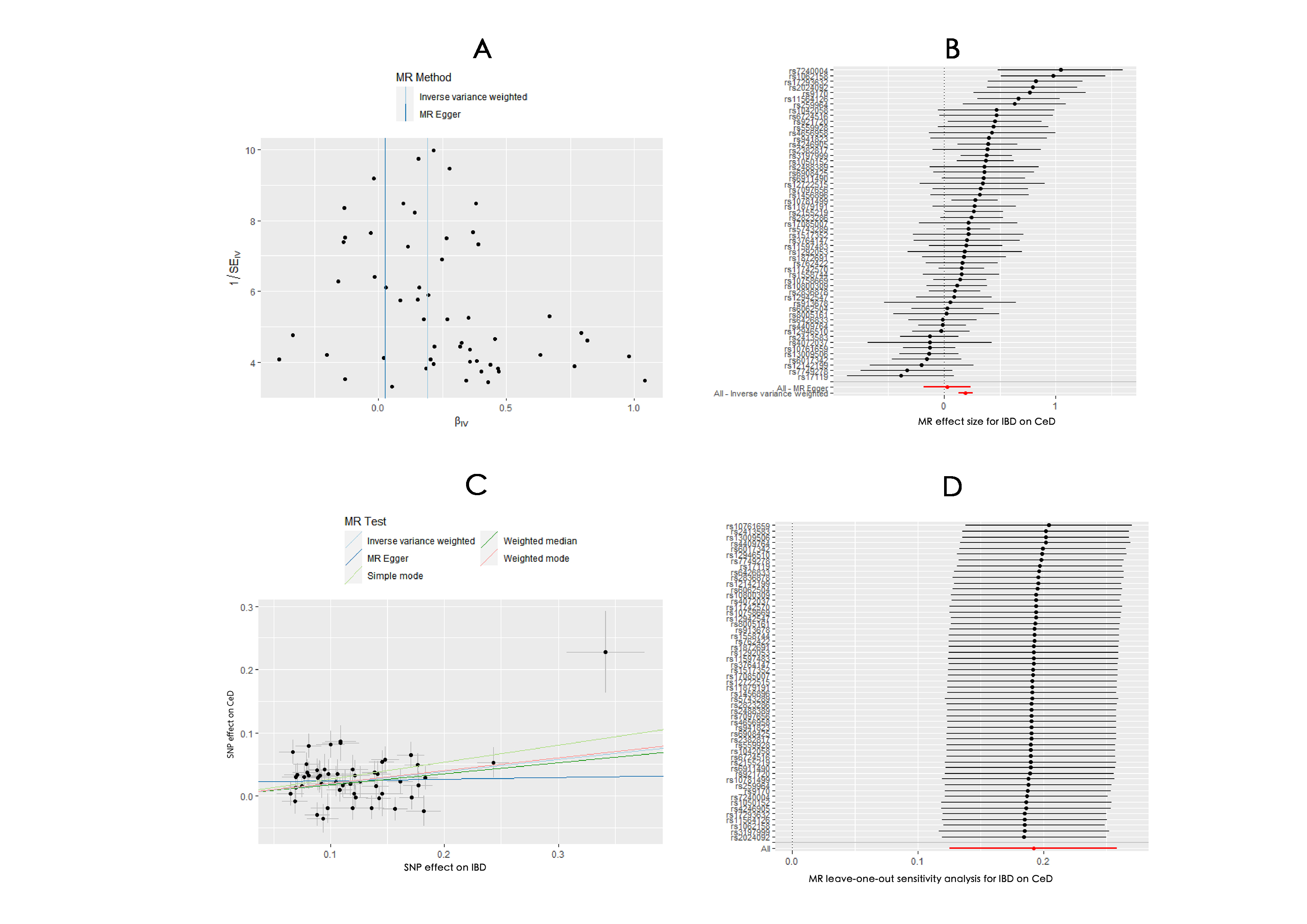


Figure S10 Funnel plot (A), forest plot (B), scatter plot (C), and leave-one-out analysis (D) of the causal effect of IBD on CeD risk.


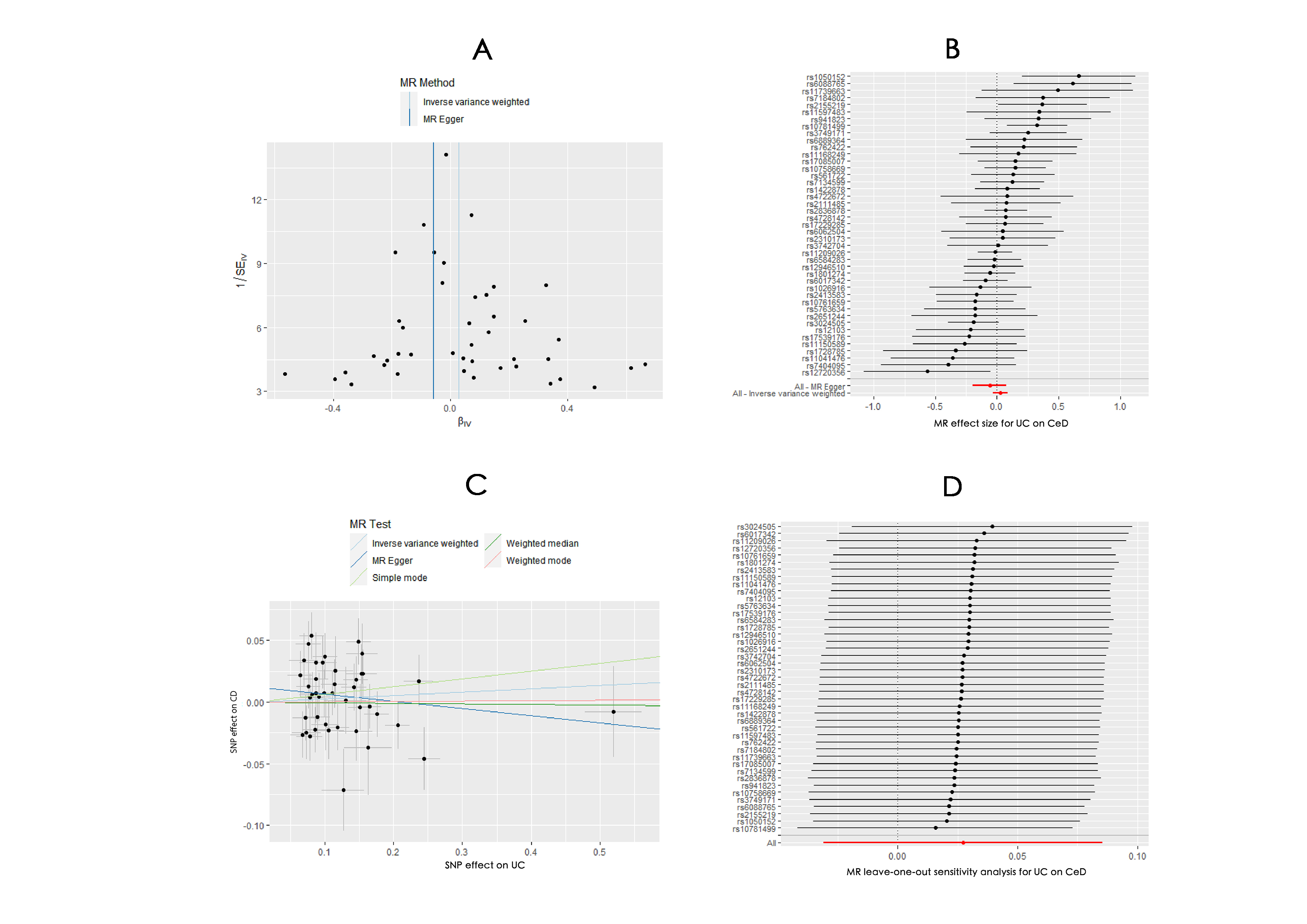


Figure S11 Funnel plot (A), forest plot (B), scatter plot (C), and leave-one-out analysis (D) of the causal effect of UC on CeD risk.


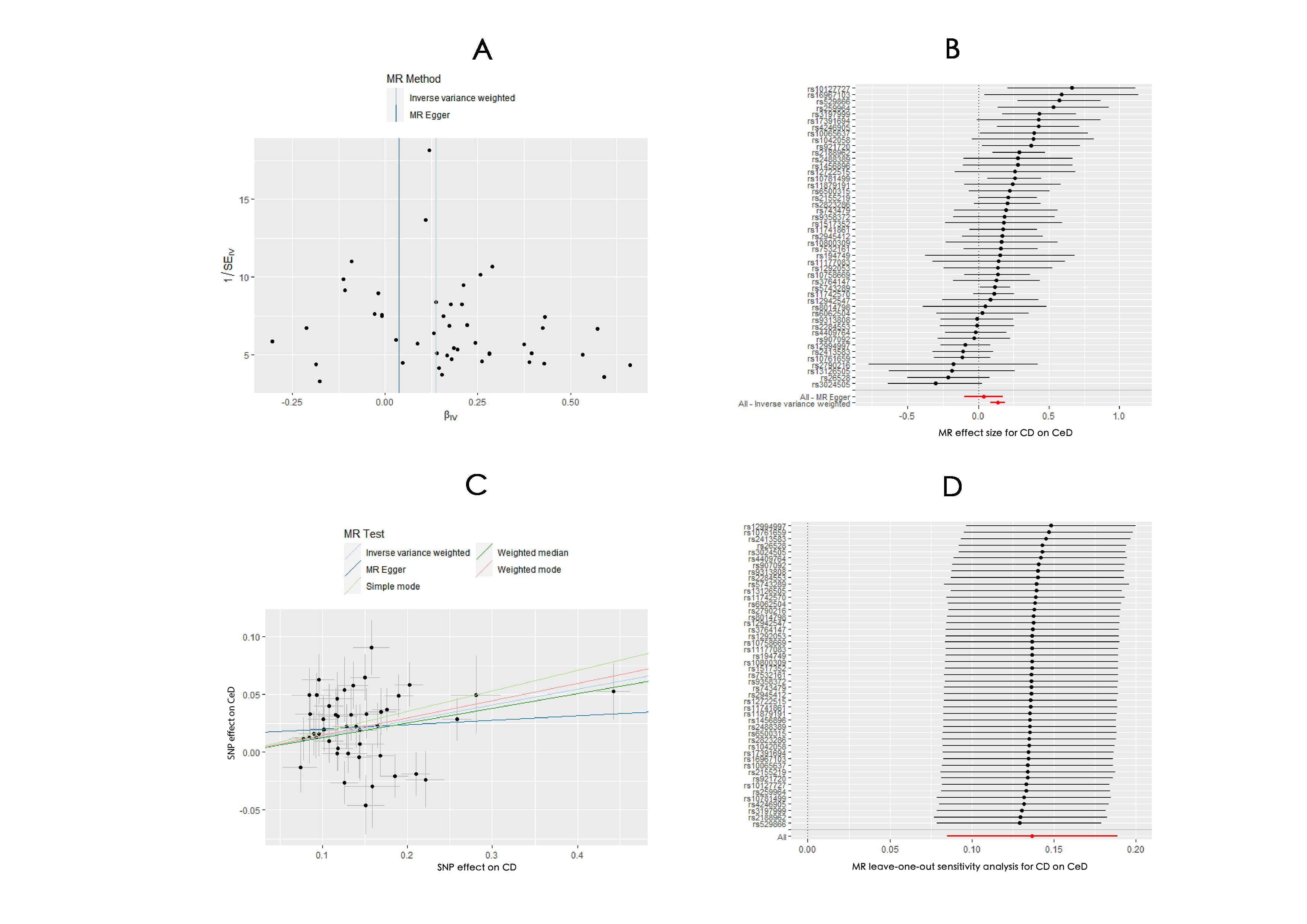


Figure S12 Funnel plot (A), forest plot (B), scatter plot (C), and leave-one-out analysis (D) of the causal effect of CD on CeD risk.


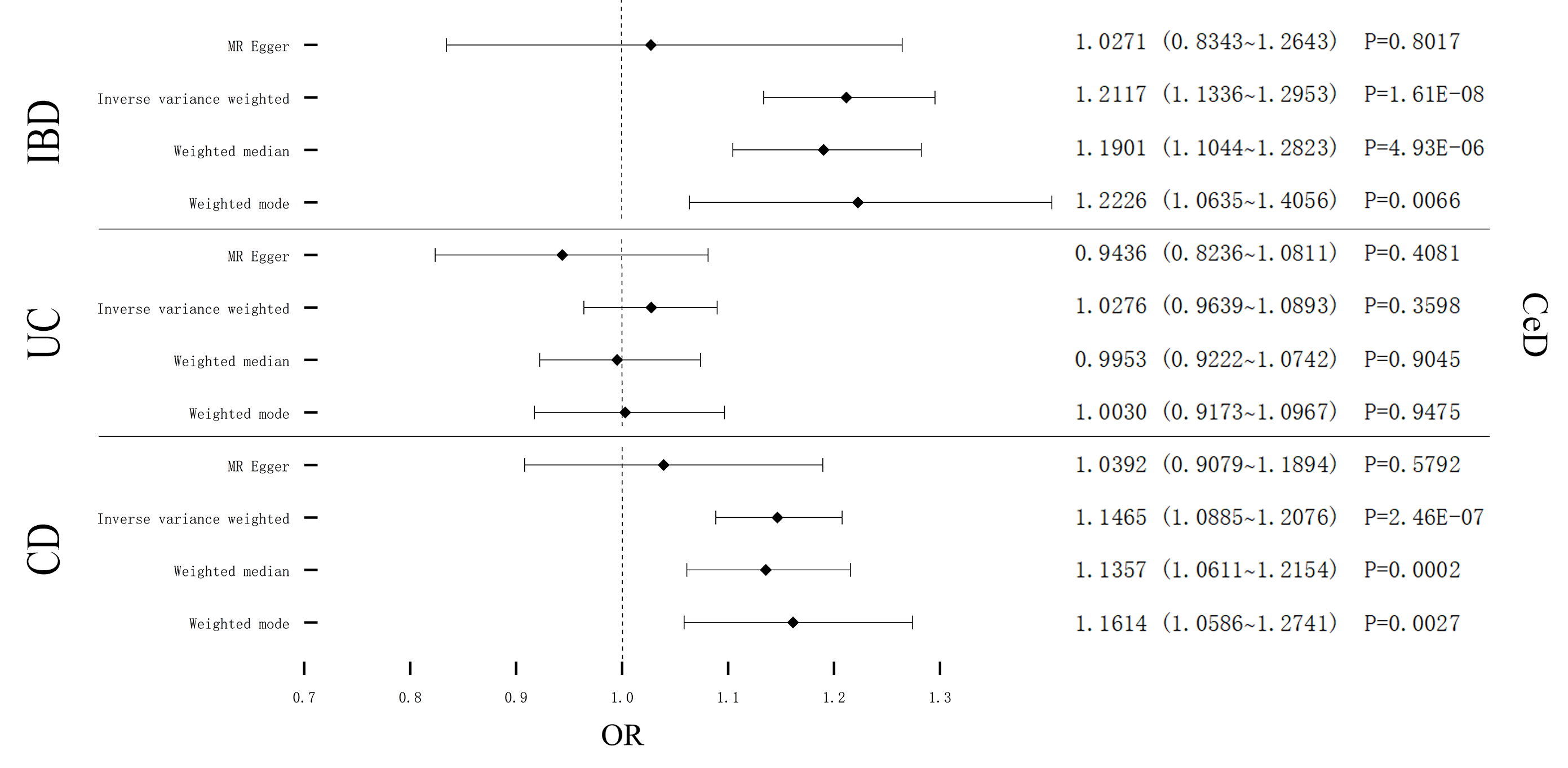


Figure S13 Causal estimates given as odds ratios (ORs) and 95% confidence intervals for the effect of Celiac Disease on inflammatory bowel disease, Ulcerative colitis and Crohn's disease. CeD, Celiac Disease; IBD, Inflammatory Bowel Disease; UC, Ulcerative Colitis; CD, Crohn's Disease.


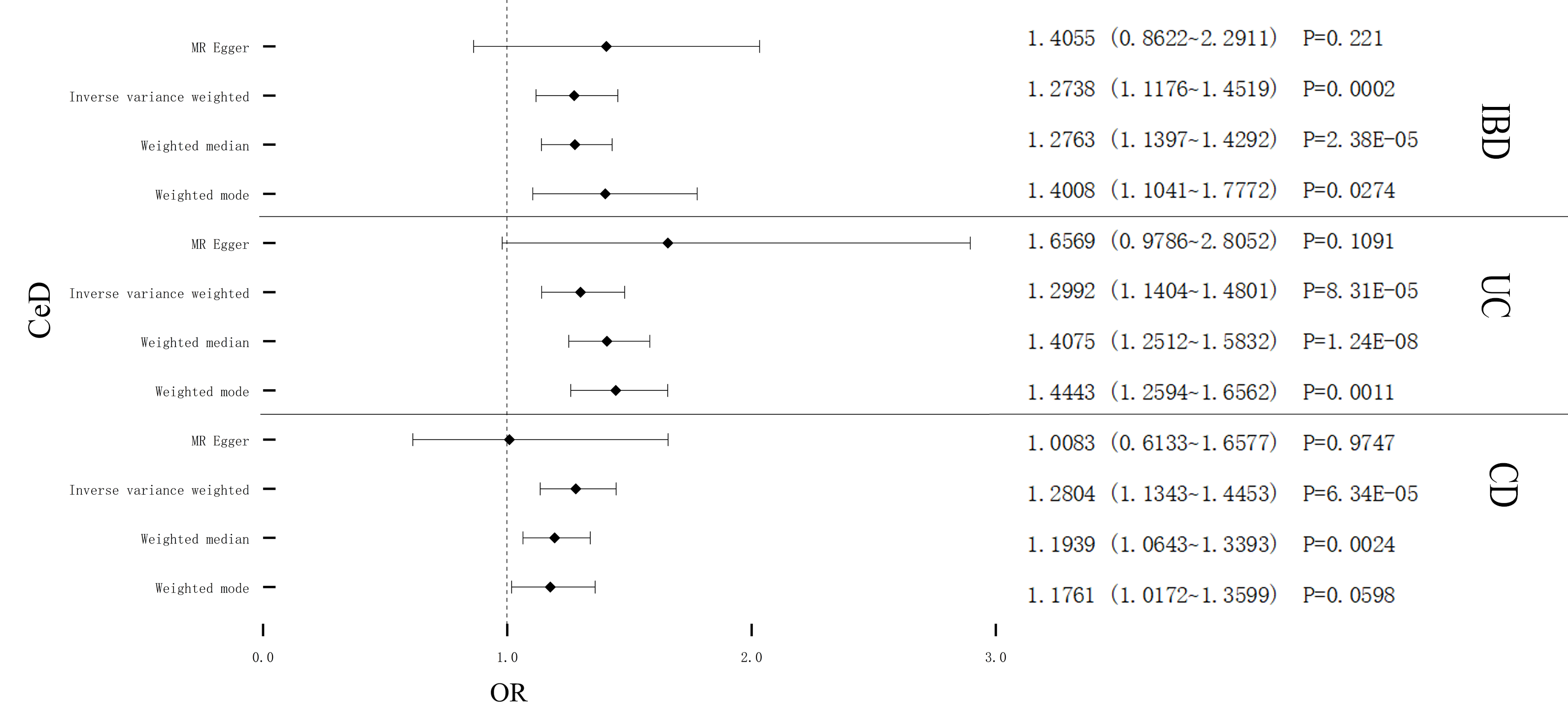


Figure S14 Causal estimates given as odds ratios (ORs) and 95% confidence intervals for the effect of inflammatory bowel disease, Ulcerative colitis and Crohn's disease on Celiac Disease. IBD, Inflammatory Bowel Disease; UC, Ulcerative Colitis; CD, Crohn's Disease; CeD, Celiac Disease
